# Interface Issues of Layered Transition Metal Oxide Cathodes for Sodium-Ion Batteries: Current Status, Recent Advances, Strategies, and Prospects

**DOI:** 10.3390/molecules29245988

**Published:** 2024-12-19

**Authors:** Yongxin Kuang, Yanxue Wu, Hangyu Zhang, Huapeng Sun

**Affiliations:** 1 School of Chemical Engineering and Light Industry, Guangdong University of Technology, Guangzhou 510006, China; kuangyongxin123@163.com (Y.K.); 2112206165@mail2.gdut.edu.cn (H.Z.); 2 Analysis and Test Center, Guangdong University of Technology, Guangzhou 510006, China; 3 Chenjiang Laboratory, School of New Energy, Chenzhou Vocational Technical College, Chenzhou 423000, China

**Keywords:** layered oxide cathodes, interface stability, sodium-ion batteries, surface engineering, ion doping, electrolyte optimization

## Abstract

Sodium-ion batteries (SIBs) hold significant promise in energy storage devices due to their low cost and abundant resources. Layered transition metal oxide cathodes (Na_x_TMO_2_, TM = Ni, Mn, Fe, etc.), owing to their high theoretical capacities and straightforward synthesis procedures, are emerging as the most promising cathode materials for SIBs. However, the practical application of the Na_x_TMO_2_ cathode is hindered by an unstable interface, causing rapid capacity decay. This work reviewed the critical factors affecting the interfacial stability and degradation mechanisms of Na_x_TMO_2_, including air sensitivity and the migration and dissolution of TM ions, which are compounded by the loss of lattice oxygen. Furthermore, the mainstream interface modification approaches for improving electrochemical performance are summarized, including element doping, surface engineering, electrolyte optimization, and so on. Finally, the future developmental directions of these layered Na_x_TMO_2_ cathodes are concluded. This review is meant to shed light on the design of superior cathodes for high-performance SIBs.

## 1. Introduction

The high consumption of traditional energy sources, such as oil and natural gas, has aggravated the global energy crisis and caused an ever-increasing demand for new energy sources. However, current new energy sources such as wind, tidal, and solar energy are intermittent. It is urgently needed to explore new energy storage devices with high efficiencies, low costs, environmental friendliness, and long lifespans. Lithium-ion batteries (LIBs), due to their high energy density and long life cycle, have dominated the secondary battery market [1,2,3]. However, their further widespread application is hindered by the limited and uneven distribution of lithium resources. Sodium-ion batteries (SIBs) emerged as a promising candidate in large energy storage fields because of their lower cost, abundant raw materials, and similar electrochemical behaviors to LIBs [4,5]. Cathode materials not only occupy a large mass fraction but also determine the cost, safety, cycling life, and energy and power density of SIBs. Up until now, various cathode materials have been explored for SIBs, including transition metal oxides (Na_x_TMO_2_, TM = Ni, Mn, Fe, etc.) [6], Prussian blue analogs (Fe_4_[Fe(CN)_6_]_3_) [7], and polyanionic compounds (Na_x_M_y_(X_a_O_b_)Z_w_) [8]. Among them, Na_x_TMO_2_ has a high specific capacity and tap density, high cost-effectiveness, and a simple manufacturing process, and holds great commercial application prospects.

Na_x_TMO_2_ can be divided into the P2-type, O3-type, and so on due to their different crystal structures. P2-type or O3-type layered Na_x_TMO_2_, owing to their high specific capacities of 180~220 mAh g^−1^, moderate operating potentials of 2.7–3.2 V (vs. Na^+^/Na), and controllable synthesis, stimulated considerable attention [9,10,11]. Nevertheless, these Na_x_TMO_2_ cathodes still struggle to meet the required capacity and life cycle in stationary energy storage systems, which can be attributed to their interfacial chemistry and mechanical instability [12,13]. The causes of bad interface problems are as follows. First, these Na_x_TMO_2_ are sensitive to air. The materials’ atmospheric exposure to H_2_O, CO_2_, and O_2_ promotes acidic and oxidative degradation, forming inactive compounds such as NaOH and NaHCO_3_ on the surface, thus weakening the interfacial stability [14,15]. Furthermore, when the operation voltage exceeds 3.5 V, the cathode experiences significant de-sodiation, leading to shifts between the sodium and TM layers [16]. Concurrently, the Jahn–Teller (J–T) effect induces distortions in some TM ions (Mn^3+^, Ni^3+^, and Fe^3+^), promoting their migration within and between layers [17]. At a higher voltage (>4.2 V), oxygen ions (O^n−^, n ≤ 2) participate in charge compensation, causing irreversible oxygen electron transfer and phase transitions and initiating structural degradation and volume strain [18]. This process is accompanied by substantial surface degradation, including cracking, roughening, and second-phase growth, resulting in an unstable cathode–electrolyte interface (CEI) [19,20]. Moreover, the electrolyte corrodes the cathode surface under high voltages, and lattice oxygen escapes from the cathode, forming gases such as CO_2_ and O_2_ [21]. Ultimately, these changes accelerate the dissolution of TM ions, further deteriorating the battery’s performance [22].

To address the challenges layered Na_x_TMO_2_ faced, various approaches have been proposed to enhance interface stability, including surface engineering, ion doping, electrolyte optimization, and so on [23,24]. These strategies could not only mitigate the degradation of Na_x_TMO_2_ cathodes under air exposure but also support the long-term degradation of the CEI during cycling [25]. One commonly used method is to construct a coating layer on the surface of layered Na_x_TMO_2_ cathodes during the material synthesis process. This approach significantly reduces the exposure of active materials to air, thereby limiting the degradation of Na_x_TMO_2_ caused by air. The coating layer could also act as a robust physical barrier to effectively avoid corrosion from the electrolyte and reduce the side reactions between the cathode and electrolyte, thus stabilizing the CEI [26,27]. Furthermore, Na_x_TMO_2_ with a coated layer could suppress the migration of TM ions and oxygen species, alleviate volume strain during cycling, and inhibit phase transitions [28,29]. As research progresses, the variety of coating materials has expanded to include metal oxides [30], metal fluorides [31], solid electrolytes [32], etc. These materials typically offer both thermodynamic and chemical stability, enabling them to withstand the harsh conditions of repeated cycling. Beyond surface coatings, Na_x_TMO_2_ cathode doping with ions (such as Mg^2+^, Al^3+^, Ti^4+^, and F^−^) could enhance TM–O bonding, resulting in a more stable layered crystal structure, thus reducing structural cracks and forming a stable interface [33,34]. Minimizing structural cracks effectively impedes electrolyte penetration and mitigates undesirable interfacial side reactions. It is worth noting that ions with different radii occupied different crystal structure sites, resulting in different influences on the Na_x_TMO_2_ cathode. For example, Ca^2+^ occupies the Na sites due to its similar radius to Na^+^, acting as a “pillar” that stabilizes the structure during Na^+^ extraction/insertion [35]. On the other hand, Li^+^, Ti^4+^, and Al^3+^ tend to integrate into the TM layer, reinforcing TM–O bonding and impeding the migration and dissolution of TM ions, thereby enhancing structural stability [36,37]. In addition, the side reaction cathode and the electrolyte also severely influence the interfacial stability of the Na_x_TMO_2_ cathode. Therefore, optimizing the electrolyte to construct a dense, uniform CEI could also improve the interfacial stability of Na_x_TMO_2_ cathodes. For example, the addition of pentafluorophenoxycyclotriphosphazene (FPPN) has been proven to create a stable inorganic fluoride CEI on Na_x_Ni_y_Fe_z_Mn_a_O_2_, preventing parasitic reactions between the cathode and the electrolyte [38].

**Figure 1:**
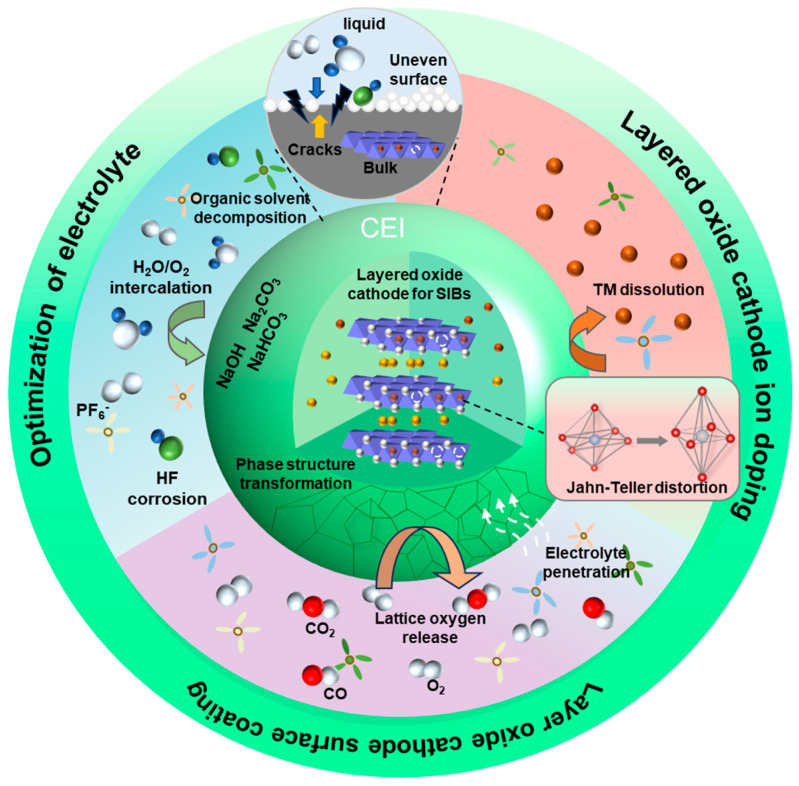
Illustrates the main factors that affect the interface stability of the layered Na_x_TMO_2_ cathode.

As illustrated in Figure 1, this review summarized the critical factors affecting the interfacial stability and degradation mechanisms of Na_x_TMO_2_, including H_2_O and CO_2_ intercalation caused by air sensitivity, the migration and dissolution of TM ions, the loss of lattice oxygen, and electrolyte corrosion. The novelty of this review lies in its systematic exploration of the interplay between interfacial stability and modification strategies for layered oxide cathodes. This work comprehensively reviewed the existing mainstream modification approaches to improving the interface stability of layered Na_x_TMO_2_, including ion doping, surface coating, and electrolyte composition optimization. Furthermore, the modification strategy to improve electrochemical performance is summarized, including element doping, surface engineering, electrolyte optimization, and so on. Finally, we put forward an interface modification strategy and the future development directions of these layered Na_x_TMO_2_ cathodes. It is hoped that this review could provide meaningful guidance on designing high-performance cathodes for SIBs.

## 2. Infrastructure Characteristics of Na_x_TMO_2_

**Figure 2:**
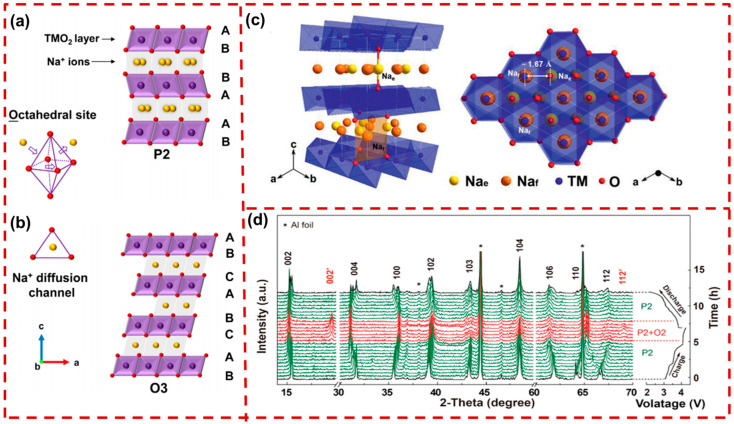
Schematic illustration of the layered Na_x_TMO_2_ crystal structures of (**a**) P2-type and (**b**) O3-type [39]. Copyright 2023, Wiley. (**c**) Structure schematic of the Na_e_ and Na_f_ sites in the typical P2-type Na_x_TMO_2_ [43]. Copyright 2021, Wiley. (**d**) In situ XRD patterns of an NNM cathode during cycling [44]. Copyright 2022, Wiley.

Based on the Na^+^ chemical environments and stacking sequences of oxygen ions, layered Na_x_TMO_2_ cathodes can be divided into the P2-type or the O3-type, where P or O indicates that the Na^+^ occupies prismatic or octahedral sites, respectively. As displayed in Figure 2a, the P2-type layered cathodes belong to the hexagonal crystal system with space group P63/mmc. The stacking of TMO_2_ and oxygen layers follows the ABBAAB pattern. The Na^+^ occupies prismatic voids at BB or AA oxygen layers, while TM ions occupy octahedral voids at AB oxygen layers, alternating to form a layered structure. In O3-type layered cathodes, Na^+^ ions reside in octahedral voids of the face-centered cubic oxygen cell, arranged with an ABCABC oxygen stacking sequence (Figure 2b) [39]. O3-type Na_x_TMO_2_ materials offer a higher Na^+^ content (0.7 < x ≤ 1), providing a high initial coulombic efficiency (ICE). When charging at high voltage, some Na^+^ deviates from the octahedral sites to energy-stable prismatic voids, together with the generation of vacancies. This process is accompanied by TMO_2_ layers sliding without destruction of the TM–O bond, resulting in the formation of the P3 phase [40]. Furthermore, additional de-intercalation will result in increasingly complicated phase transitions of O3→O′3→P3→P′3→P3”, causing high stress and structural degradation, thereby influencing the cycling performance and rate capability [41]. Even less fortunately, Na^+^ escaping from one octahedral site to another necessitates traversing a tetrahedral position, which increases the diffusion barrier, ultimately causing a sluggish diffusion kinetic in O3-type Na_x_TMO_2_ [42].

Compared to O3-type Na_x_TMO_2_, P2-type Na_x_TMO_2_ with a low Na^+^ content (0.3 < x < 0.7) resulted in a low ICE. When charging to a higher voltage, the Na^+^ escapes and the P2 phase generally transforms into the O2 phase structure due to the gliding of TMO_2_ layers, causing considerable structural shrinkage and shortened interlayer spacing. Despite the fact that Na^+^ can move in a straight line via the two coplanar prismatic sites, contributing to a higher diffusion rate of Na^+^ ions, the P2 phase structure generally suffers from Na^+^/vacancy and charge ordering structures, which reduce its diffusion rate. As shown in Figure 2c, Na^+^ ions in P2-type layered oxides occupy two different sites with trigonal prism coordination—the Na_f_ (0, 0, 1/4) and Na_e_ (1/3, 2/3, 1/4) sites. Limited by the short distance between adjacent Na_e_ and Na_f_ sites (≈1.67 Å), Na^+^ ions (with an ionic diameter ≈ 2.04 Å) are not allowed to occupy these two nearest-neighboring sites simultaneously, leading to ordered Na^+^/vacancies [43]. For example, as exhibited in Figure 2d, the P2-Na_2/3_Ni_1/3_Mn_2/3_O_2_ (NNM) cathode undergoes a P2–O2 phase transition due to anion redox reactions at high voltages, resulting in lattice strain and surface cracking [44]. In both O3- and P2-type cathodes, high-voltage-induced phase transitions lead to repeated volume strain, causing mechanical failure in particles and surface cracking. These phase transitions and fractures severely compromise the structural integrity of the CEI, accelerating electrolyte infiltration and TM ion dissolution.

## 3. Current Interface Issues for Na_x_TMO_2_

In recent years, layered Na_x_TMO_2_ materials have garnered extensive attention due to their low cost, simple synthesis, and similar manufacturing process to LIBs’ ternary oxide cathode. However, serial factors such as air-induced degradation, irreversible phase transition under high voltages, and electrolyte corrosion accelerate the failure of the Na_x_TMO_2_ cathode. These factors all involve the interface stability of the Na_x_TMO_2_ cathode. The degradation mechanisms affecting interface stability are still not clear.

### 3.1. Air Stability

The interaction between the complex components in air and Na_x_TMO_2_ has a significant impact on the structural stability of the surface-to-bulk. This interaction leads to the formation of substances such as NaOH, Na_2_CO_3_, and NiO on the surface of the Na_x_TMO_2_, severely affecting the interface stability.

**Figure 3:**
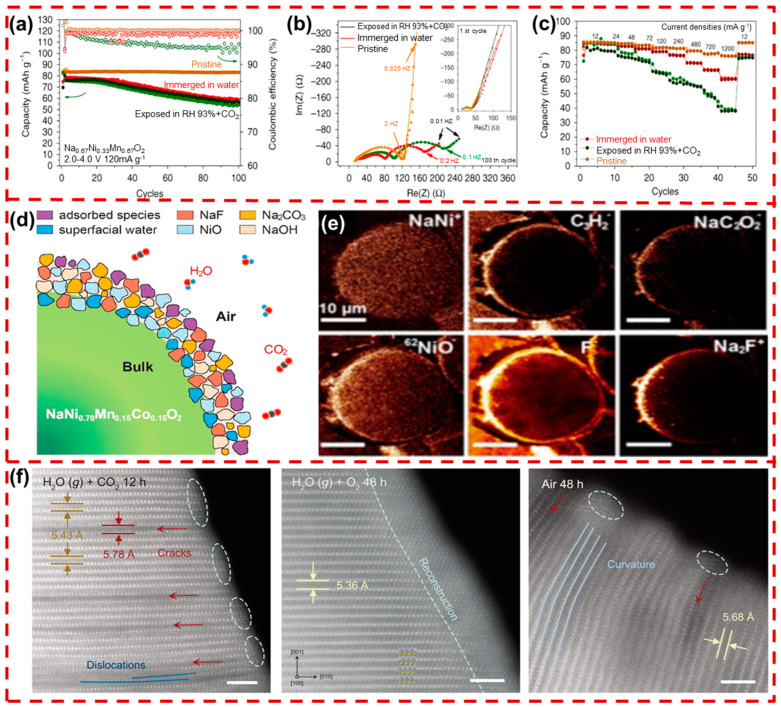
(**a**) The cycling performance of Na_0.67_Ni_0.33_Mn_0.67_O_2_ at 120 mA g^−1^, (**b**) comparison of the impedance (at the 1st and 100th cycles with frequencies from 100 kHz to 10 MHz), and (**c**) the rate capability of Na_0.67_Ni_0.33_Mn_0.67_O_2_ exposure to three environments (pristine, RH 93% and CO_2_, and water) [46]. Copyright 2020, Springer Nature. (**d**) Chemical model of NaNi_0.7_Mn_0.15_Co_0.15_O_2_ exposed to air, (**e**) cross-sectional TOF-SIMS mapping of NaNi_0.7_Mn_0.15_Co_0.15_O_2_ exposed to air for 24 h, showing the distribution of various secondary ions, including NaNi^+^, C_3_H^2–^, NaC_2_O_2_^2–^, ^62^NiO^–^, F^–^, and Na_2_F^+^ fragments [49]. Copyright 2018, American Chemical Society. (**f**) STEM images of NFM111 stored in CO_2_ with water vapor for 12 h, O_2_ with water vapor for 48 h, and moist air (RH = 60%, CO_2_ concentration ~600 ppm) for 48 h [51]. Copyright 2024, AAAS.

The origin of alkaline compounds on the cathode surface is quite complex. On the one hand, Na^+^ deposited on the surface during material sintering quickly reacts with air components, forming residual surface alkali (NaOH, Na_2_CO_3_, and NaHCO_3_). At the same time, H_2_O intercalates into the cathode, with CO_2_ promoting proton exchange between Na^+^ and H^+^, generating Na_2_CO_3_ and NaHCO_3_ (acidic degradation). Additionally, the coexisting H_2_O and O_2_ can oxidize certain TM ions in Na_x_TMO_2_, resulting in the formation of NaOH (oxidative degradation) [45]. This phenomenon not only leads to Na^+^ loss, but the resulting surface alkaline compounds also cause severe interfacial side reactions and performance degradation. As shown in Figure 3a–c, Na_0.67_Ni_0.33_Mn_0.67_O_2_ (NNM) exhibited significant capacity fade and increased electrochemical impedance after exposure to air. Interface layer breakage allows electrolyte penetration, further damaging the cathode’s internal structure [46]. The presence of residual alkali accelerates electrolyte consumption and significantly increases impedance due to their insulating nature [47]. Due to the hygroscopic nature of NaOH, it can absorb large amounts of water, which further promotes the reaction of CO_2_ on the cathode particles [48]. Time-of-flight secondary ion mass spectrometry (TOF-SIMS) results (Figure 3e) reveal a substantial accumulation of F^−^ on the surface of NaNi_0.7_Mn_0.15_Co_0.15_O_2_ particles after exposure to air for 24 h. This phenomenon illustrates that NaNi_0.7_Mn_0.15_Co_0.15_O_2_ exposure to air results in surface alkaline compounds such as NaOH and Na_2_CO_3_, triggering PVDF defluorination and NaF formation (Figure 3d) [49].

Apart from the alkaline surface residual, the surface reconstruction caused by air degradation also presents a major challenge to the interface stability of Na_x_TMO_2_. During acidic degradation, the presence of CO_2_ disrupts the balance of Na^+^/H^+^ exchange, leading to proton exchange. This process causes uneven Na^+^ extraction and induces the surface reduction of Mn^3+^ or Mn^4+^ [50]. The uneven extraction and reduction destabilize the cathode’s original surface structure, resulting in interlayer bending and the formation of structural cracks, which compromise the layered framework. In contrast to acid degradation, oxidative degradation acts on both the surface and the bulk. It interacts with unstable TM ions, such as Ni^3+^, through the co-existing H_2_O and O_2_, reducing them to the rock-salt phase (NiO, etc.). As shown in high-resolution scanning transmission electron microscopy (STEM) images of the NaNi_1/3_Fe_1/3_Mn_1/3_O_2_ (NFM111) cathode (Figure 3f), the air degradation leads to the formation of rock-salt structures and surface cracks, gradually spreading into the bulk [51]. The intercalation of H_2_O also expands the Na layer’s interlayer spacing, forming a structurally unstable hydrated phase [52]. Air causes irreversible damage to the surface structure of the Na_x_TMO_2_ cathode, meaning that it is necessary to protect the cathode interface and reduce the exposed surface area.

### 3.2. Dissolution and Migration of the Jahn–Teller Effect in Transition Metal Oxides

In Na_x_TMO_2_ cathodes, TM ions with the Jahn–Teller (J–T) effect typically migrate or dissolve into the electrolyte due to the instability of their electronic structure. The migration results in defect vacancies of TM ions on the cathode, triggering the collapse of the original structure on the Na_x_TMO_2_ cathode and the formation of surface cracks [53]. The dissolution of TM ions from the CEI catalyzes electrolyte decomposition, forming a high-impedance and unstable interface on the cathode surface. Meanwhile, the dissolved TM ions are driven by concentration gradients and electric forces, thickening the SEI and hindering Na^+^ transport [54,55,56].

High-valence TM ions (Mn^4+^, Fe^5+^, and Ni^4+^) are strong oxidizing agents that promote electrolyte decomposition and oxidize electrolyte solvent molecules into CO_2_ and other reactive substances through redox pathways [57]. Meanwhile, TM ions are reduced to stable low-valence states (Mn^2+^, Fe^3+^, and Ni^2+^) and produce fluoride attached to the interface. Furthermore, the Na_x_TMO_2_ contains TM ions with J–T activity (Mn^3+^, Ni^3+^, etc.), which are prone to J–T distortions, leading to irreversible phase transitions (from cubic to tetragonal) and crack formation during cycling. The distortion of the crystal structure also facilitates the dissolution of TM ions into the electrolyte [58]. To more clearly explain the dissolution and migration mechanism of active J–T, several different TM ions were selected for discussion.

**Figure 4:**
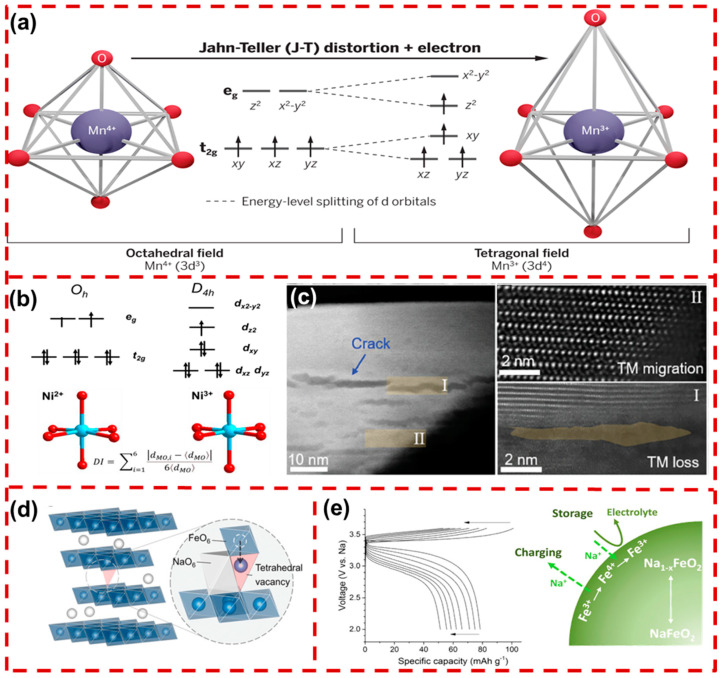
(**a**) The ligand field in manganese oxides distorts the six equivalent metal–oxygen bonds (**left**) into two longer axial bonds and four shorter equatorial bonds (**right**) [62]. Copyright 2020, AAAS. (**b**) The migration pathway of Ni^3+^ from an octahedron to a tetrahedron in layered oxides, (**c**) TEM-HAADF image and its enlarged Region I and Region II of cycled NFM [64]. Copyright 2020, Wiley. (**d**) Illustration of a possible migration pathway of iron [67]. Copyright 2023, Wiley. (**e**) The Fe^4+^/Fe^3+^ redox model at the interface indicates that the reduction of Fe^4+^ is coupled with the oxidation of electrolytes, thereby increasing the interfacial impedance [68]. Copyright 2015, American Chemical Society.

Some studies have found that when PF_6_^−^ is used as the electrolyte, Mn ions dissolve in the form of Mn^3+^ and Mn^2+^ [59,60,61]. Mn^2+^ accelerates the decomposition of the electrolyte and generates side reaction products such as hydrofluoric acid (HF), which could corrode the CEI and Na_x_TMO_2_. Furthermore, cathodes containing Mn^3+^ have a higher dissolution rate than those of other TM ions because of the higher J–T activity. As illustrated in Figure 4a, Mn^4+^ can remain relatively stable in an octahedral structure within the TMO_2_. After the J–T effect occurs on Mn^3+^, the electron distribution in the high-spin e_g_ orbital is not uniform. To eliminate degeneracy, two axial TM–O bonds are extended while the other bonds contract, distorting the original structure [62]. The J–T distortion results in longer TM–O bonds with lower 2p orbital overlap, and the O on the longer TM–O bonds possesses a higher negative charge, thereby enhancing its Lewis base strength and promoting reactions between Mn^3+^ and the generated HF, leading to their dissolution into the electrolyte. Simultaneously, the structural distortions cause obvious volume changes and irreversible phase transitions, resulting in the attenuation of battery capacity [63].

Similar to Mn^3+^, Ni^3+^ is also affected by octahedral distortions. Within an O3- Na_0.987_Ni_0.396_Fe_0.204_Mn_0.402_O_2_ (NFM) material, as the content of Ni^3+^ increases and its orbital degeneracy gradually rises, J–T distortion will occur to eliminate this degeneracy and stabilize the structure (Figure 4b). The J–T distortion causes anisotropic changes in the Ni–O bonds, promoting the migration of TM ions. Due to the sliding of the TMO_2_ slabs and the propagation of anisotropic strain, severe cracks and lattice defects are generated in NFM (Figure 4c). The formation of cracks exposes more surface area to the electrolyte, intensifying side reactions, which in turn leads to the fragmentation and degradation of NFM [64].

Recent research indicates that the strong interaction between Fe^3+^ and oxygen ions in the FeO_6_ octahedral facilitates redox reactions that generate Fe^4+^ [65]. Under a high voltage, iron-based oxides form an octahedral Fe^4+^-O_6_ structure with cation vacancies, exhibiting significant J–T distortion. As Na^+^ is extracted during charging, vacancies accumulate in the Na layers, prompting Fe ions to migrate into these layers. This migration increases “vacancy-O-vacancy” configurations, further initiating redox reactions among oxide ions. However, this migration is only partially reversible, and active oxygen readily escapes from the structure, undermining material stability [66]. When the Fe content in the cathode exceeds one-third, this phenomenon becomes more obvious. The migration diagram in Figure 4d shows Fe^3+^ migrating from octahedral sites in TMO_2_ to the Na layers. After a certain amount of Na^+^ extraction, the activation energy for Fe^3+^ migration from its original octahedral position significantly decreases [67]. This promotes Fe^3+^ migration, ultimately affecting Na^+^ transport. Meanwhile, the highly oxidative Fe^4+^ can also promote the decomposition of carbonate-based electrolytes, leading to the formation of an unstable CEI (Figure 4e) [68].

The migration and dissolution of TM ions impact the structural and interface stability of the entire Na_x_TMO_2_ cathode. Therefore, investigating the mechanisms of the J–T effect will help guide the interface modifications of the CEI, thus enhancing the structural stability and reducing the dissolution and migration of TM ions.

### 3.3. Phase Structure Evolution

Na_x_TMO_2_ undergoes complex phase structural changes during charging/discharging, caused by the release of lattice oxygen [69], the migration of TM ions [70], and the interlayer O-O repulsion force [71]. Stress and volume expansion originating from phase transitions accelerate the formation of surface cracks and lead to surface structural degradation. This results in promoting side reactions between the electrolyte and the cathode, thereby affecting the stability of the CEI and ultimately deteriorating electrochemical performance. It is well-recognized that the Na_x_TMO_2_ cathode surface will preferentially transition from a layered structure to a spinel-like or rock-salt structure, resulting in voltage attenuation [72]. The spinel-like and rock-salt structures will gradually propagate from the material surface to the interior of the bulk, leading to a continuous discharge voltage platform. To explain the mechanism more clearly, the following describes the phase transition of P2- and O3-type Na_x_TMO_2_ materials.

**Figure 5:**
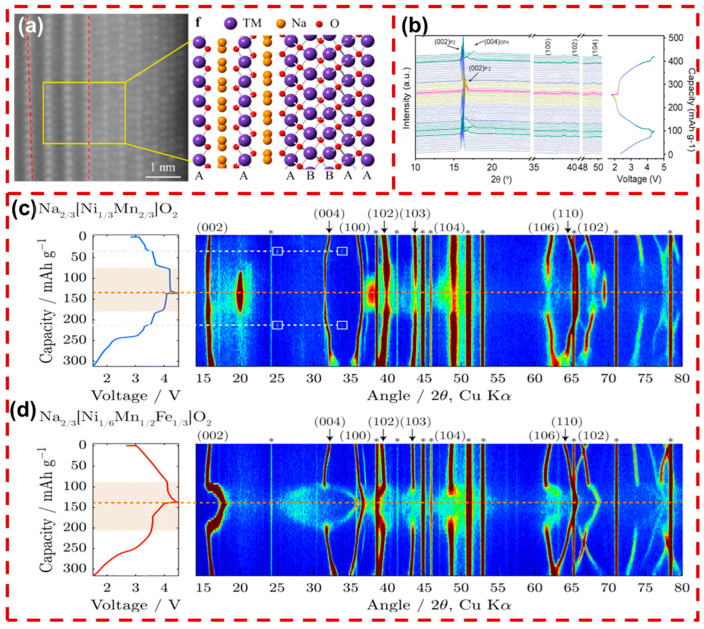
(**a**) Observing intragranular cracks in Na_2/3_Ni_1/3_Mn_2/3_O_2_ samples after 50 cycles at 2.0–4.25 V with STEM, which shows the atomic configurations of the phase boundary [74]. Copyright 2018, Elsevier. (**b**) In situ XRD patterns of the P2-Na_0.706_MnO_2_ during the initial two charges/discharges [75]. Copyright 2021, Springer Nature. (**c**) Initial cycle’s operando XRD maps of Na_2/3_Ni_1/3_Mn_2/3_O_2_ and (**d**) Na_2/3_[Ni_1/6_Mn_1/2_Fe_1/3_]O_2_ [76]. Asterisks (*) denote reflections associated with the operando cell window and inactive electrode constituents (e.g., carbon). Copyright 2019, Royal Society of Chemistry.

P2-type Na_x_TMO_2_ with a large Na interlayer spacing facilitates Na^+^ transport [73]. P2-type Na_x_TMO_2_ undergoes phase transitions to O2 or OP4 structures during charging, with the formation of a P’2’ (signifies monoclinic distortion) phase during discharging. Generally, phase transitions are accompanied by crack formation and surface reconstruction. As shown in Figure 5a, the surface of P2-Na_2/3_Ni_1/3_Mn_2/3_O_2_ (P2-NNM) undergoes reconstruction after cycling, resulting in TM ions in a stacking structure that is distinct from the original P2 phase. The phase transition leads to TM ions being inserted into the Na layer, forming a stacking sequence of the “AABB” [74]. As displayed in Figure 5b, the crystal structure and lattice parameters of P2-Na_0.706_MnO_2_ (P2-NM) undergo significant changes during charging, resulting in the formation of an OP4 phase (consisting of alternating P2–O2–P2 layers) [75]. This P2–OP4 phase transition is reversible and causes less volumetric change compared to the P2–O2 transition. As depicted in Figure 5c, Na_2/3_Ni_1/3_Mn_2/3_O_2_ experienced a P2–O2 transition with phase boundaries due to substantial differences between the two phases, which led to surface cracks in particles. In contrast, P2-Na_2/3_[Ni_1/6_Mn_1/2_Fe_1/3_]O_2_ (P2-NFM) does not possess a superlattice structure and undergoes a shift from an ordered to a disordered Na^+^/vacancy arrangement, resulting in a P2–OP4 phase transition without forming phase boundaries [76]. This smoother transition mitigates structural deformation, helping to suppress lattice expansion and thereby reducing the likelihood of surface cracking.

During discharging, the intercalation of Na^+^ leads to a similar P2–P’2 phase transition in P2 Na_x_TMO_2_. It is well-known that Na content is a critical factor for the structural stability of the P2-Na_x_TMO_2_. However, the deintercalation of Na^+^ reduces the shielding effect between TMO_2_ layers, prompting the movement of TMO_2_ layers and leading to structural collapse [77]. Therefore, retaining more Na^+^ in the P2-type material helps to maintain structural stability during charging/discharging. Currently, many studies are focusing on synthesizing more stable P’2-type Na_x_TMO_2_ with a high Na^+^ content via ion doping [78,79].

**Figure 6:**
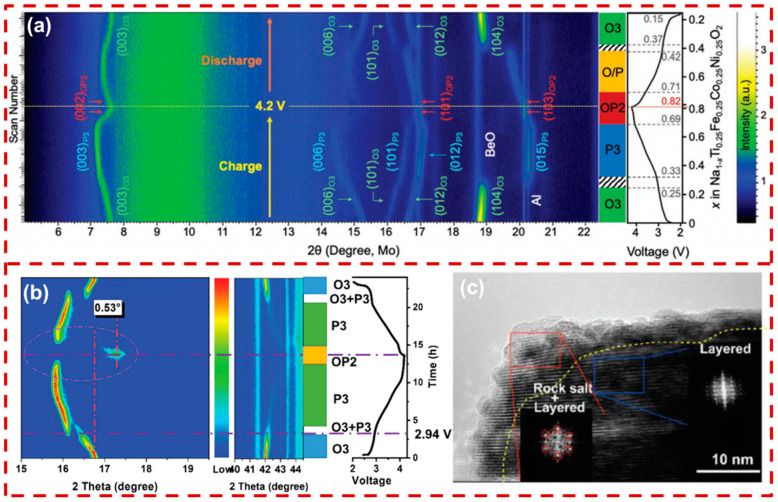
(**a**) An intensity contour plot of the in situ XRD peaks for Na_1−x_TFCN in 2~4.2 V [81]. Copyright 2020, Wiley. (**b**) In situ XRD patterns evolution of (003) and (104) diffraction peaks, as well as corresponding charge and discharge curves, for NFM, and (**c**) HRTEM images and FFT (inset) patterns of NFM. The rock-salt phase is marked by a red rectangle [82]. Copyright 2023, Wiley.

As Na^+^ is extracted during the charging process, numerous prismatic vacancies cause the O3 phase to be transferred to a P3 phase (oxygen stacking sequence (“ABBCCA”)). The O3 phase transitions to a lattice-distorted O’3 phase during discharging [80]. Figure 6a illustrates that as Na^+^ is extracted, the Na interlayer’s spacing increases, causing expansion along the c-axis and shortening the TM–O bond lengths, triggering a phase transition from O3 to P3. At a high voltage above 4 V, the collapse of Na layers induces the formation of a new OP2 phase [81]. The O–P phase evolution in layered oxide cathodes is commonly linked to c-axis expansion. Specifically, as shown in Figure 6b, the O3-Na_0.8_Ni_0.4_Fe_0.2_Mn_0.4_O_2_ (O3-NFM) cathode undergoes significant c-axis contraction during high-voltage charging, resulting in the emergence of a new OP2 phase. This phase transition leads to volumetric changes that generate surface cracking. Furthermore, redox reactions between TM ions and the organic electrolyte at the CEI release lattice oxygen, which accelerates surface reconstruction. As illustrated in Figure 6c, this structural degradation disrupts the layered structure, leading to the formation of a rock-salt phase that impedes Na^+^ diffusion [82]. The surface reconstruction causes irreversible damage to the CEI, thereby increasing side reactions and hindering Na^+^ transport. Therefore, mitigating phase transitions of O3-type Na_x_TMO_2_ materials is essential for maintaining cathode surface integrity, reducing the dissolution and migration of TM ions at the interface, and improving the structural stability of the CEI.

### 3.4. Influence of Electrolytes

Organic liquid electrolytes have been widely used because of their excellent ion conductivity and low resistivity. However, the electrolyte component can react with active substances in Na_x_TMO_2_ to form the CEI during cycling, which greatly determines the battery’s performance. Common interfacial side reactions for Na_x_TMO_2_ include HF attacks due to the salt decomposition of sodium hexafluorophosphate (NaPF_6_) [83,84]. These phenomena not only deplete the electrolyte but also lead to CEI reconstruction, increasing interfacial impedance. For the layered Na_x_TMO_2_, persistent interfacial side reactions will result in a loose rock-salt structure on the cathode surface, accelerating the dissolution of TM ions and the release of lattice oxygen.

#### 3.4.1. Cathode–Electrolyte Interface (CEI)

The cathode–electrolyte interphase (CEI) plays a critical role in the electrochemical performance and stability of layered Na_x_TMO_2_. During the initial charging, electrolyte molecules decompose at the cathode surface and form the CEI, and its long-term stability is essential to ensure consistent battery operation [85]. While this CEI layer mitigates further electrolyte decomposition, it often demonstrates instability in the early stages of operation. This instability of the CEI increases charge transfer resistance during cycling, impeding the diffusion of Na^+^ ions and subsequently reducing cycle stability [86,87].

From a chemical and structural perspective, solvation and desolvation processes are pivotal to the formation and evolution of the CEI layer on the cathode surface [88]. The solvation structure is primarily governed by the coordination of Na^+^ with electronegative atoms in solvent molecules (e.g., carbonyl or ether oxygens) or anions. The binding energy of these interactions significantly depends on the types of cations, anions, and solvents involved [89]. Moreover, impurities of the electrolyte, unintended reaction by-products, and specific operational conditions will affect the CEI layer, further compromising the cathode’s performance [90,91].

Interfacial side reactions occur between the electrolyte and the exposed cathode surface during cycling, leading to the thickening of the CEI due to the accumulation of by-products [92]. Furthermore, high-voltage operation exacerbates the decomposition of electrolytes, generating corrosive substances such as HF. These substances could erode the CEI, causing more surface exposure and promoting the further dissolution of TM ions [93,94]. The stability of the CEI is intricately linked to the chemical properties of the electrolyte, including the types of solvents, salts, and additives [95,96,97]. The composition and stability of the electrolyte during polarization largely dictate the CEI’s properties, which in turn affect the battery’s performance and longevity. Consequently, electrolyte engineering emerges as a highly effective strategy for optimizing CEI stability and mitigating adverse interfacial reactions [98].

#### 3.4.2. Interfacial Side Reaction

**Figure 7:**
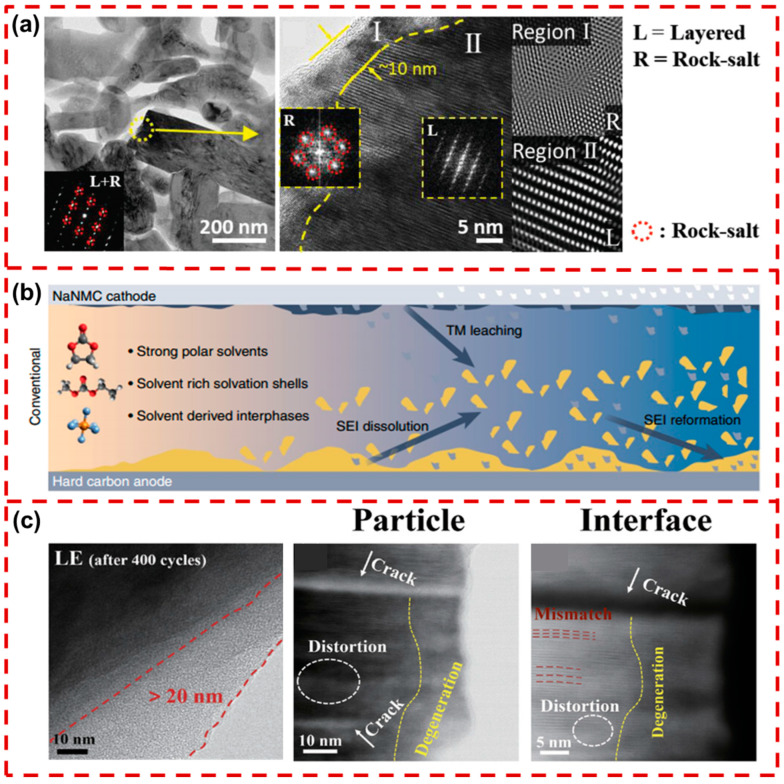
(**a**) Bright-field TEM images of a primary particle at the core (the fully discharged Na[Ni_0.5_Mn_0.5_]O_2_ cathode after 100 cycles at the upper cut-off voltage of 4.0 V), with a SAED pattern (inset) (weaker additional spots from the rock-salt phase are marked by red circles) [99]. Copyright 2020, Wiley. (**b**) The structural diagram of the dissolution of TM ions at the CEI and catalytic reforming in the SEI, utilizing NaNi_0.68_Mn_0.22_Co_0.1_O_2_ as the cathode material [105]. Copyright 2022, Springer Nature. (**c**) The STEM images of NLNM after 400 cycles using the LE electrolyte [106]. Copyright 2023, Wiley.

As previously noted, the emergence of micro-cracks on the surface of Na_x_TMO_2_ cathodes is associated with the phase transitions and migration of TM ions. These cracks increase the exposed surface area, facilitating the penetration of electrolytes. Under the continuous decomposition of electrolytes, the cathode’s surface structure undergoes reorganization, resulting in an uneven CEI layer. As shown in Figure 7a, the surface of Na[Ni_0.5_Mn_0.5_]O_2_ cathode particles is severely degraded by the electrolyte, leading to the formation of a rock-salt phase structure (NiO) [99]. This phenomenon indicates that highly oxidative TM ions (Ni^4+^, Fe^4+^) in primary particles are susceptible to surface degradation when in contact with the electrolyte under deep de-sodiation conditions. Simultaneously, interfacial side reactions are accompanied by the dissolution of TM ions and the release of oxygen, severely impacting the life cycle of the layered Na_x_TMO_2_.

The electrolyte not only oxidizes the active materials but also reacts with residual alkalis on the cathode surface, inducing the formation of uneven CEI films and CO_2_ during cycling [100,101]. For instance, during the cycling of P2-type Na_0.85_Li_0.12_Ni_0.22_Mn_0.66_O_2_ (P2-NLNM), the electrolyte EC (ethylene carbonate) reacts with surface Na_2_CO_3_, resulting in the release of CO_2_ and O_2_. The side reactions can be described as follows [102]:

2Na_2_CO_3_ − 4e^−^ → 2CO_2_↑+ O_2_↑+ 4Na^+^ (1)

O^2−^ + EC + Na^+^ − 2e^−^ → ROCO_2_Na + H_2_O + CO2↑+ CO↑ + O_2_↑ (2)

Continuous gas production will cause volume expansion, which may lead to internal structural misalignment, ultimately affecting battery performance and lifespan. In addition, residual trace water and high-temperature conditions can promote the decomposition of NaPF_6_, producing acidic compounds such as HF and PF_5_. PF_5_ also reacts with H_2_O to form POF_3_, which damages the crystal structure of Na_x_TMO_2_. These by-products attack Na_x_TMO_2_’s surface, not only degrading the CEI but also accelerating the dissolution of TM ions [103,104]. As illustrated in Figure 7b, the interaction between the cathode and the electrolyte leads to a persistent dissolution of TM ions and subsequent structural degradation of the CEI [105].

The stability of the Na_x_TMO_2_’s surface is critical for achieving a stable CEI and thereby superior cycling performance. As shown in Figure 7c, the P2/O3-Na_0.67_Li_0.16_Ni_0.33_Mn_0.67_O_2+δ_ (NLNM) cathode was observed to exhibit an excessively thick CEI layer (~15 nm) after 400 cycles in a 1M NaPF_6_ liquid electrolyte (LE). Additionally, numerous intragranular cracks were detected in the NLNM particles, along with dense lattice mismatches and surface defects, which significantly hinder Na^+^ transport [106]. This behavior suggests that the interface side reaction promotes electrolyte decomposition and cathode surface reconstruction, leading to the dissolution of TM ions and the formation of intragranular cracks. Addressing the interface side reaction is essential for enhancing the cycling stability of the layered Na_x_TMO_2_ cathode.

## 4. Recent Advances and Strategies for the Interface Issues of Na_x_TMO_2_

The electrochemical performance of SIBs is largely influenced by the interface stability of the Na_x_TMO_2_ cathodes. It was explored that several factors contribute to the degradation of the Na_x_TMO_2_ cathode interface, including the intercalation of H_2_O, O_2_, and CO_2_ due to air exposure, surface cracking resulting from high-voltage irreversible phase transitions, and interfacial side reactions between the cathode and electrolyte. To mitigate these issues and enhance the interface stability of the cathode, various modification strategies are employed, such as coating [107], ion doping [108], and electrolyte modification [109]. These interfacial modifications strengthen the cathode’s surface structure, prevent undesirable phase transitions, effectively inhibit side reactions, and limit the dissolution of transition metal ions. Herein, recent research on interface modification of layered Na_x_TMO_2_ cathodes was reviewed.

### 4.1. Phase Structure Evolution

Surface coating has been proven a highly effective method of enhancing the stability of layered cathode interfaces. Up to now, many coating materials have been explored, including metal oxides, phosphates, borates, and fluorides. Various coating techniques, such as wet coating, dry coating, and chemical vapor deposition, have been developed to further expand the range of coating options. These coatings on the surface of the Na_x_TMO_2_ cathode remove alkaline surface residuals and serve as physical barriers to increase stability to air. Furthermore, it could avoid direct contact with the electrolyte, inhibiting interfacial side reactions and thus protecting the cathode material during cycling, thereby enhancing stability and performance [110,111].

#### 4.1.1. Metal Oxide Coatings

**Figure 8:**
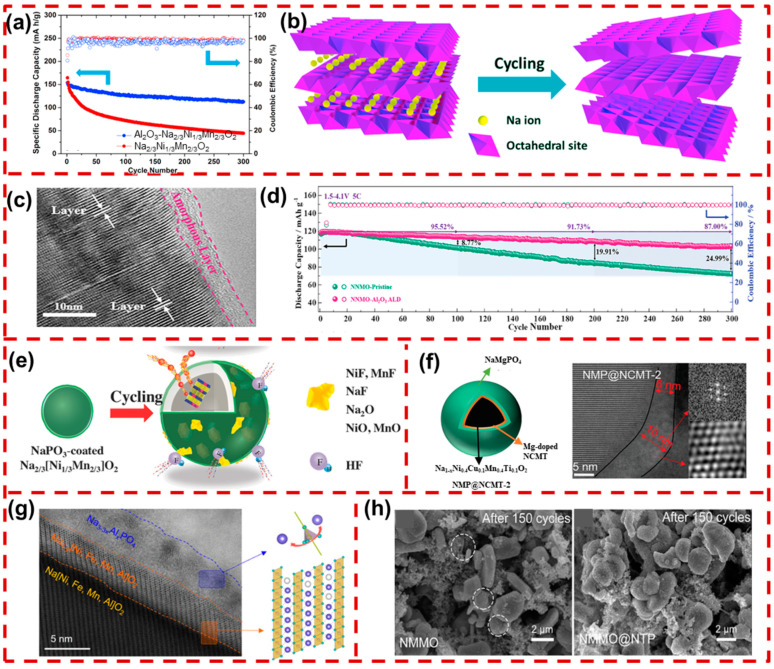
(**a**) Comparison of the electrochemical properties of P2-NNM and P2-Al_2_O_3_-NNM. (**b**) Schematic figure of exfoliation during the sodiation and de-sodiation processes of the P2-NNM particle [114]. Copyright 2016, Elsevier. (**c**) Ex situ HRTEM images of particles (P2/P3-ALD-Al_2_O_3_-NNM) at the interface, (**d**) long-term cycling test of P2/P3-NNM-Pristine and P2/P3-ALD-Al_2_O_3_-NNM at 5 C [116]. Copyright 2021, Wiley. (**e**) Schematic illustration of by-products on the surface of NaPO_3_-coated NNM (after 50 cycles) [129]. Copyright 2018, Wiley. (**f**) Structural schematic and STEM images of NMP@NCMT [130]. Copyright 2023, Wiley. (**g**) STEM image of a particle cross-section of modified Na_3–3x_Al_x_PO_4_ [132]. Copyright 2023, American Chemical Society. (**h**) SEM images of P2-NNMO and P2-NMMO@NTP after 150 cycles at 0.2 C [133]. Copyright 2020, Elsevier.

In recent years, researchers have devoted their efforts to constructing metal oxide coating layers to improve the interface stability of Na_x_TMO_2_ cathodes, including Al_2_O_3_, ZrO_2_, TiO_2_, SnO_2_, and In_2_O_3_. These metal oxides could act as physical barriers between the cathode material and the electrolyte without participating in the electrochemical reaction. Their superior mechanical rigidity and electrochemical stability help mitigate the stress caused by volume changes during cycling [112,113]. For instance, Zhou et al. applied an Al_2_O_3_ coating on the surface of P2-type Na_2/3_[Ni_1/3_Mn_2/3_]O_2_, significantly improving cyclic stability and achieving an enhanced 73.2% retention after 300 cycles (Figure 8a). Furthermore, as illustrated in Figure 8b, the uncoated material undergoes a P2–O2 phase transition under high voltages, resulting in volume expansion and single-crystal particle spalling. The Al_2_O_3_ coatings not only suppressed interfacial side reactions but also stabilized the crystal structure, thus preventing structural degradation under high-voltage cycling [114]. Kalapsazova et al. effectively improved the cycling stability of Na_2/3_Ni_1/2_Mn_1/2_O_2_ (NNM) through Al^3+^ doping and an Al_2_O_3_ coating. The Al_2_O_3_ coating successfully suppressed interfacial side reactions, preserving the structural integrity and stability of the material [115]. To achieve a uniform and thin coating, Ji’s group synthesized an Al_2_O_3_-coated P2/P3-Na_2/3_Ni_1/3_Mn_2/3_O_2_ (P2/P3-NNM) via an atomic layer deposition (ALD) method. It can be observed from the TEM image (Figure 8c) that a homogeneous Al_2_O_3_ film was intact on the surface of P2/P3-Al_2_O_3_-NNM after 100 cycles. The ALD-induced Al_2_O_3_ coatings effectively mitigate stress accumulation from volume changes, helping to stabilize the interface structure of P2/P3-NNM. As shown in Figure 8d, even at a high current density of 5C, the P2/P3-Al_2_O_3_-NNM maintained a capacity of 105 mAh·g^−1^ after 300 cycles with a capacity retention rate of 87.0%, which is much higher than that of pristine P2/P3-NNM (25%) [116]. Compared to traditional sol-gel and hydrothermal methods, (ALD) creates uniform and high-quality coating layers. This nanoscale surface coating does not hinder Na^+^ diffusion or impact the material structure [117,118].

ZrO_2_ has also proven to be an effective coating material for layered oxide cathodes [119,120]. Hence, Liu et al. coated ZrO_2_ on the O3-type NaMn_1/3_Fe_1/3_Ni_1/3_O_2_ and found that it could reduce electrolyte corrosion, inhibit Na_2_CO_3_ formation, and suppress CO_2_ release. XRD analysis further revealed that Zr^4+^ doped into the TM sites strengthened the bond between Zr^4+^ and O^2−^, which enhanced the structural stability of the TMO_2_ layer [121]. Yang et al. designed ZnO coatings on P2-Na_2/3_[Ni_1/3_Mn_2/3_]O_2_ and found that they could maintain particle integrity and inhibit side reactions at the cathode–electrolyte interface [122]. Additionally, Sun’s group found that coating MgO on O3-Na[Ni_0.5_Mn_0.5_]O_2_ (O3-NNM) could significantly improve electrochemical performance. The MgO coatings protect the cathode surface from liquid electrolyte corrosion, particularly from HF. Additionally, some Mg^2+^ doped in the crystal structure replaced Ni^2+^ in the TM sites, which suppressed irreversible polyphase transitions during cycling [123]. Liu et al. demonstrated that coating TiO_2_ on P2-Na_0.85_Li_0.12_Ni_0.22_Mn_0.66_O_2_ (P2-NLNMO) significantly improved both cycling stability and rate performance. Their work revealed that the lattice oxygen content of TiO_2_-coated NLNMO not only did not decrease but significantly increased. This indicates that the TiO_2_ coating prevented interfacial side reactions, maintaining the structural integrity and stability. Additionally, some Ti^4+^ ions were incorporated into the cathode lattice, which expanded the TMO_6_ octahedra and increased the interlayer spacing, thereby enhancing Na^+^ diffusion kinetics [124]. Zhang et al. applied a SnO_2_ coating on the P2-Na_0.6_[Li_0.2_Mn_0.8_]O_2_ (P2-NLMO) via a liquid-phase method. The coated SnO_2_ notably improved the rate performance of NLMO due to its ability to provide a stable ion transport interface, facilitating Na^+^ transport [125]. Moreover, the SnO_2_ coating helps prevent lattice oxygen loss under high voltages, enhancing the effective utilization of anionic redox reactions and improving cycling reversibility [126,127]. Kalapsazova et al. found that coating P3-Na_2/3_Ni_1/2_Mn_1/2_O_2_ (P3-NNM) with CeO_2_ is an effective strategy to enhance cycling stability and rate performance. Through electrochemical and ex situ electron paramagnetic resonance (EPR) experiments, they demonstrated that CeO_2_ improves the redox activity of oxygen, contributing to increased capacity [128]. These coating strategies create stable barriers that protect the cathode’s internal structure from air and electrolyte corrosion, suppress irreversible phase transitions, and mitigate the volume change, thus improving cycling stability.

#### 4.1.2. Coatings Made from Other Materials 

In addition to metal oxides, phosphates, metal fluorides, and solid electrolytes have also been reported as coating layers for Na_x_TMO_2_. Jo et al. synthesized a NaPO_3_ layer to coat the surface of P2-Na_2/3_[Ni_1/3_Mn_2/3_]O_2_ (P2-NNM). The NaPO_3_ coating layer effectively blocks HF and H_2_O released from the decomposition of NaPF_6_ on the P2-NNM surface during the cycling process. As illustrated in Figure 8e, the NaPO_3_ coating shell scavenged HF and thus decreased the content of HF and H_2_O molecules in the electrolyte, which could effectively suppress the detachment of the active materials, ensuring superior cycling stability. Additionally, TOF-SIMS characterization of the NNM coated with NaPO_3_ revealed a significant reduction in the intensities of peaks corresponding to NaCO^+^, NaC^+^, and Na_2_OH^+^ compared with that of pristine NNM, demonstrating the effective removal of surface residual alkali [129].

During the sintering process, Na^+^ tends to accumulate on the surface of Na_x_TMO_2_ materials, leading to the formation of residual alkaline compounds (NaOH, Na_2_CO_3_, and NaHCO_3_). These compounds could induce interfacial side reactions and degrade the electrochemical performance of Na_x_TMO_2_. To address this issue, Hu et al. transformed the residual alkalis into a solid electrolyte coated on the surface of O3-NaNi_0.4_Cu_0.1_Mn_0.4_Ti_0.1_O_2_ (O3-NCMT) to stabilize the interface. As illustrated in Figure 8f, the addition of Mg(CH_3_COO)_2_ and H_3_PO_4_ leads to reactions with the surface residues, resulting in the formation of a NaMgPO_4_ (NMP) coating layer on the surface of O3-NCMT. The detailed reaction mechanism is outlined below:

3Na^+^ + PO_4_^3−^ → Na_3_PO_4_ (3)

Na^+^ + Mg^2+^ +PO_4_^3−^ →NaMgPO_4_ (4)

3Mg^2+^ + 2PO_4_^3−^ → Mg_3_(PO_4_)_2_ (5)

The designed NaMgPO_4_ coating layer not only effectively removed the residual alkali on the NCMT surface, but also created a specialized ion-conductive pathway that facilitates Na^+^ transport [130]. Jo et al. developed a β-NaCaPO_4_ coating nanolayer on the P2-Na_2/3_[Ni_1/3_Mn_2/3_]O_2_ by reacting CaHPO_4_ and Ca_2_P_2_O_7_ with surface residues during the synthesis process. This approach eliminates residual surface alkali and leverages the strong binding affinity of Ca^2+^ and PO_4_^3−^ to enhance the stability of the interfacial layer. TOF-SIMS and HF titration results demonstrated that the β-NaCaPO_4_ coating effectively scavenges HF and water molecules from the electrolyte, thereby inhibiting electrolyte decomposition [131]. Hu’s group developed an in situ plastic crystal Na_3–3x_Al_x_PO_4_ coated on O3-NaNi_0.4_Fe_0.2_Mn_0.4_O_2_ (O3-NFM424) using a simple one-step process. As shown in Figure 8g, the STEM images reveal a distinct Na_3–3x_Al_x_PO_4_ layer with a thickness of 3–4 nm covered on the particle surface. Furthermore, a layer of a Na-deficient phase with a small amount of a rock-salt phase between the two orange lines can be observed, which may be associated with the deep Na^+^ extraction during the formation of the Na_3–3x_Al_x_PO_4_ layer. This Na_3–3x_Al_x_PO_4_ layer not only eliminates residual alkali on the NFM424 surface but also acts as a protective barrier against the parasitic reactions between the cathode and electrolyte [132]. Wang et al. employed a dual modification strategy by combining Mg substitution with a NASICON-type NaTi_2_(PO_4_)_3_ (NTP) coating to enhance the cycle stability and rate performance of P2-Na_0.67_Ni_0.33_Mn_0.67_O_2_ (P2-NNMO). As shown in Figure 8h, the SEM images of NTP-Na_0.67_Ni_0.28_Mg_0.05_Mn_0.67_O_2_ (NMMO) maintained a relatively intact morphology after 150 cycles, without visible cracking observed, in contrast to the original NNMO particles. Electrochemical impedance spectroscopy (EIS) and the constant current intermittent titration technique (GITT) also demonstrated that NTP-NNMO exhibits a stable structure with high Na^+^ conductivity [133]. Liu’s group also confirmed that an NTP coating on the surface of P2-Na_0.65_[Mn_0.70_Ni_0.16_Co_0.14_]O_2_ can effectively suppress lattice volume shrinkage and P2–O2 phase transition. The doping of Ti^4+^ can act as a pillar to inhibit TMO_2_ sliding. Moreover, the highly electronegative PO_4_^3−^ can substitute oxygen sites, tightly bond with transition metals, and stabilize the structure [134].

The designed NaMgPO_4_ coating layer not only effectively removed the residual alkali on the NCMT surface, but also created a specialized ion-conductive pathway that facilitates Na^+^ transport [130]. Jo et al. developed a β-NaCaPO_4_ nanolayer coating on P2-Na_2/3_[Ni_1/3_Mn_2/3_]O_2_ by reacting CaHPO_4_ and Ca_2_P_2_O_7_ with surface residues during the synthesis process. This approach eliminates surface alkali residues and leverages the strong binding affinity of Ca^2+^ and PO_4_^3−^ to enhance the stability of the interfacial layer. TOF-SIMS and HF titration results demonstrated that the β-NaCaPO_4_ coating effectively scavenges HF and water molecules from the electrolyte, thereby inhibiting electrolyte decomposition [131]. Hu’s group developed an in situ plastic crystal Na_3–3x_Al_x_PO_4_ coated on O3-NaNi_0.4_Fe_0.2_Mn_0.4_O_2_ (O3-NFM424) using a simple one-step process. As shown in Figure 8g, the STEM images reveal a distinct Na_3–3x_Al_x_PO_4_ layer with a thickness of 3–4 nm covered on the particle surface. Furthermore, a layer of a Na-deficient phase with a small amount of a rock-salt phase between the two orange lines can be observed, which may be associated with the deep Na^+^ extraction during the formation of the Na_3–3x_Al_x_PO_4_ layer. This Na_3–3x_Al_x_PO_4_ layer not only eliminates residual alkali on the NFM424 surface but also acts as a protective barrier against the parasitic reactions between the cathode and electrolyte [132]. Wang et al. employed a dual modification strategy by combining Mg substitution with a NASICON-type NaTi_2_(PO_4_)_3_ (NTP) coating to enhance the cycle stability and rate performance of P2-Na_0.67_Ni_0.33_Mn_0.67_O_2_ (P2-NNMO). As shown in Figure 8h, the SEM images of NTP-Na_0.67_Ni_0.28_Mg_0.05_Mn_0.67_O_2_ (NMMO) maintained a relatively intact morphology after 150 cycles, without visible cracking observed, in contrast to the original NNMO particles. Electrochemical impedance spectroscopy (EIS) and the constant current intermittent titration technique (GITT) also demonstrated that NTP-NNMO exhibits a stable structure with high Na^+^ conductivity [133]. Liu’s group also confirmed that an NTP coating on the surface of P2-Na_0.65_[Mn_0.70_Ni_0.16_Co_0.14_]O_2_ can effectively suppress lattice volume shrinkage and P2–O2 phase transition. The doping of Ti^4+^ can act as a pillar to inhibit TMO_2_ sliding. Moreover, the highly electronegative PO_4_^3−^ can substitute oxygen sites, tightly bond with transition metals, and stabilize the structure [134].

Except for phosphates, metal fluorides have also achieved promising results as surface coatings layer for Na_x_TMO_2_. Sun et al. applied an AlF_3_ layer coated on the O3-Na[Ni_x_Co_y_Mn_z_]O_2_ (O3-NCM) microsphere, which consists of nanorods. High-resolution TEM (HRTEM) results after cycles revealed that the AlF_3_ coatings effectively mitigated volume expansion during phase transitions, thereby preventing the formation of micro-cracks. Moreover, SEM and XRD analyses confirmed that AlF_3_ coating significantly hinders electrolyte penetration, contributing to enhanced structural integrity [135]. Wen et al. developed a spinel-Co_x_B dual protective layer on the surface of NaNi_1/3_Fe_1/3_Mn_1/3_O_2_ (NFM) using a liquid-phase reduction method at room temperature. In situ XRD observations revealed that the spinel-Co_x_B coating effectively mitigated lattice shrinkage, maintaining structural integrity during charging/discharging. Additionally, galvanostatic intermittent titration technique (GITT) measurements indicated that the Co_x_B coating layer enhanced the Na^+^ diffusion coefficient compared to pristine NFM. These improvements are attributed to the transformation of Co_x_B into a fast ionic conductor upon interaction with Na^+^ [136]. Chen and colleagues designed an ultra-thin layer of perfluorodecyltrimethoxysilane (PFDTMS) hydrophobic molecules and applied it to O3-Na(Ni_1/3_Fe_1/3_Mn_1/3_)O_2_ (O3-NFM). Their work revealed that the thickness of surface degradation products on bare O3-NFM increased sharply within a short period of air exposure, whereas this phenomenon was effectively suppressed after coating. The hydrophobic PFDTMS layer effectively prevents direct contact between the O3-NFM and the ambient air, thereby mitigating stoichiometric changes and Na/TM disorder-induced structural degradation during air storage [137].

### 4.2. Ion Doping

Na_x_TMO_2_ cathodes suffer serious phase transformations during high-voltage charging/discharging, inducing the formation of disordered spinel and rock-salt phases on the surface. These transformations are often accompanied by the release of oxygen from the lattice and the dissolution of TM ions, resulting in the generation of micro-cracks and degradation of the CEI. The resulting instability exposes more active sites to direct contact with the electrolyte, which in turn accelerates the corrosion of the cathode and decomposition of the electrolyte [138]. To address these issues, ion doping has been recognized as an effective strategy to mitigate complex phase transitions, relieve lattice strain, and stabilize TM ions [139].

**Figure 9:**
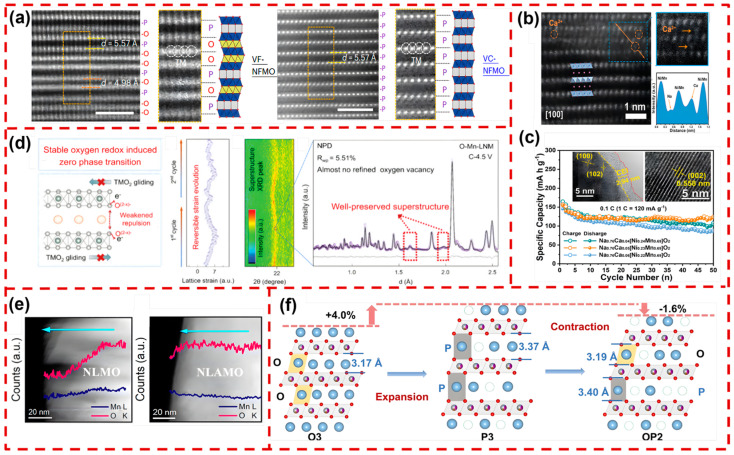
(**a**) HAADF-STEM images of VF-NFMO and VC-NFMO charged to 4.5 V taken along the (210) projection [142]. Copyright 2024, Springer Nature. (**b**) HAADF-STEM image of the P2-Na_0.76_Ca_0.05_[Ni_0.23_□_0.08_Mn_0.69_]O_2_ cathode. (**c**) Cycling performance of P2-Na_0.76_Ca_0.05_[Ni_0.23_□_0.08_Mn_0.69_]O_2_ at 0.1 C in comparison with the Ca-0.04 and Ca-0.06 counterparts (inset is the HRTEM images after 50 cycles) [143]. Copyright 2021, Wiley. (**d**) From left to right, the schematic illustration of zero-phase transition upon cycling induced by the stable oxygen redox, the lattice strain’s evolution of the O-Mn-LNM cathode during cycling processes, the well-preserved superstructure XRD during cycling, and the NPD data for the 4.5 V charged O-Mn-LNM cathode [146]. Copyright 2024, Wiley. (**e**) STEM image and EELS quantification of the O and Mn for the 10th-charged NLMO and NLAMO [147]. Copyright 2023, Royal Soc Chemistry. (**f**) Schematic illustrations of the structure change of ZT-NFMO during Na^+^ extraction [148]. Copyright 2024, Wiley.

After several decades of development, various dopant ions have been explored, including Ca^2+^, Al^3+^, Mg^2+^, Li^+^, Zn^2+^, Ti^4+^, and so on. Depending on their ionic radii, these ions can be incorporated into different sites within the Na_x_TMO_2_ crystal structure (either TM sites or Na sites). For example, Sui et al. doped Mg^2+^ into the TM site and synthesized a P2-Na_0.67_Ni_0.33_Mn_0.67_O_2_ (P2-NNM) cathode. In situ XRD analysis revealed that this Mg-doped P2-NNM undergoes a P2–OP4 phase transition while successfully suppressing the formation of the O2 phase. SEM images after cycles showed that the Mg-doped P2-NNM effectively prevented the formation of surface cracks [140]. Wang et al. employed a solid-phase synthesis method for P2-type Na_0.67_Ni_0.33_Mn_0.67_O_2_ (P2-NNM) doped with Ti^4+^. The XRD refinement results indicated that Ti^4+^ substitution caused an expanded lattice plane spacing due to its larger ionic radius compared to Mn^4+^. The enhanced Ti–O bonding promoted contraction within the TMO_2_ layer and expansion of the Na layer, which stabilized the crystal structure. Additionally, Ti^4+^ doping induced the formation of a new Z phase, effectively reducing the irreversible P2–O2 phase transition and thus enhancing electrochemical performance [141]. Yu et al. introduced uniformly dispersed fixed vacancies (denoted as “□”) in the TM layer of vacancy-free Na_0.7_Fe_0.33_Mn_0.67_O_2_ (VF-NFMO). As shown in Figure 9a, high-angle annular dark-field scanning transmission electron microscopy (HAADF-STEM) of VF-NFMO revealed distinct O-type and P-type layer stacking after charging to 4.5 V. However, the vacancy-modified Na_0.7_Fe_0.1_Mn_0.75_□_0.15_O_2_ (VC-NFMO) maintained its original P-type structure. In situ XRD confirmed that VC-NFMO effectively suppressed the P2–O2 phase transition, resulting in a smooth lattice evolution and superior interface stability during charging/discharging. This improvement is attributed to the fixed vacancies, which anchor adjacent Na^+^ and stabilize both TM and Na layers, thus preserving the structural integrity even after Na^+^ deintercalation [142]. Furthermore, the interface stability of Na_x_TMO_2_ can also be synergistically enhanced by ion doping and TM vacancy. For instance, Qu et al. developed a Ca^2^^+^ and TM vacancy-doped unique P2-Na_0.76_Ca_0.05_[Ni_0.23_□_0.08_Mn_0.69_]O_2_ cathode. The XRD refinement and HAADF-STEM analysis (Figure 9b) revealed that Ca^2^^+^ substituted some Na sites due to their similar ionic radii to Na^+^, while □ were randomly distributed at the TM sites. The HRTEM image (inset of Figure 9c) confirmed that the internal layered structure of P2-Na_0.76_Ca_0.05_[Ni_0.23_□_0.08_Mn_0.69_]O_2_ remained intact after 50 cycles with a stable and uniform CEI on the surface. Furthermore, the P2-Na_0.76_Ca_0.05_[Ni_0.23_□_0.08_Mn_0.69_]O_2_ retained a reversible capacity of 120.1 mAh g^−1^ after 50 cycles at 0.1C in the voltage range of 2.0~4.3 V (Figure 9c). The Ca^2^^+^ within the Na layer acted as “pillars”, disrupting Na^+^/vacancy ordering during charging/discharging, thus enhancing structural stability. Additionally, the “Ca-O-□” configuration strengthens bonding, effectively buffering O-O repulsion in deeply desodiated states, which mitigates the undesirable P2–O2 phase transition [143]. Chen et al. synthesized P2-Na_0.66_Li_0.18_Fe_0.12_Mn_0.7_O_2_ (P2-NLFM) by co-doping Li and Fe ions. In situ XRD results indicated that P2-NLFM maintains its phase stability without transitioning into the O2 or OP4 phase, demonstrating robust structural integrity during the cycling process [144]. Additionally, Sun’s team developed a P2-Na_0.67_Li_0.1_Fe_0.37_Mn_0.53_O_2_ (P2-NLFMO) cathode with a unique Na-O-Li configuration. Through in situ XRD and HAADF-STEM analysis, the emergence of an OP4 co-phase structure was observed under high-voltage conditions (>4.3 V). The migration of Li^+^ was found to promote lattice oxygen oxidation, with oxygen vacancies tending to concentrate around the TM layer vacancies, thus inducing a P2–OP4 phase transformation [145]. Excessive oxygen oxidation leads to molecular O_2_ formation and causes particle surface fractures, compromising electrochemical performance. To address the issue of oxygen ion over-oxidation, Zhao et al. designed a novel layered P2-Na_7/9_Li_1/9_Ni_1/9_Mg_1/9_Mn_6/9_O_2_ (O-Mn-LNM) cathode material. As shown in Figure 9d, in situ XRD refinement demonstrated that the lattice volume change for O-Mn-LNM was limited to just 0.5%, indicating quasi-zero strain characteristics during the cycling process. Ex situ neutron diffraction (NPD) data coupled with theoretical calculations further confirmed that the migration of TM ions was effectively suppressed during charging/discharging. During the desodiation process of the O-Mn-LNM cathode, the oxidation of the oxygen anion leads to the formation of O^(2−x)−^ (x denotes the charge transfer on the oxygen ion). Unlike traditional O^2−^, these O^(2−x)−^ species reduce the electrostatic repulsion between adjacent TMO_2_ layers, which helps inhibit sliding, thereby stabilizing the layered structure during cycling [146]. Chen et al. addressed lattice oxygen release by inducing Al^3+^ in Li^+^-doped Na_0.8_Li_0.24_Mn_0.85_O_2_ (NLMO). In situ differential electrochemical mass spectrometry (DEMS) analysis revealed the absence of O_2_ release peaks in the Al^3+^-doped NLMO (NLAMO), indicating suppressed oxygen evolution. As depicted in Figure 9e, doping with Al^3+^ significantly reduced the formation of surface cracks during repeated cycling. Furthermore, electron energy loss spectroscopy (EELS) exhibited uniform oxygen content across the surface and bulk of NLAMO, suggesting negligible lattice oxygen loss [147]. The complex phase transitions (O3-O’3-P3-O1) and J–T distortions of Ni^3+^ result in microcracks and surface rearrangement of the Na_x_MO_2_ cathode. To address this, Li et al. introduced Zn and Ti as co-dopants in the synthesis of O3-Na_0.9_Ni_0.4_Fe_0.1_Mn_0.5_O_2_ (O3-NFMO). As shown in Figure 9f, Zn doping facilitated the formation of a coexisting P/O phase and resulted in lattice contraction under high voltages, which mitigates expansion during Na^+^ extraction. Concurrently, Ti doping suppressed the J–T effect of Ni^3^^+^, alleviating the dissolution of TM ions during cycling [148]. Additionally, Li et al. explored the impact of Mg and Sc as dopants in P2-Na_0.67_Ni_0.33_Mn_0.67_O_2_. Resonant inelastic X-ray scattering (RIXS), pair distribution function (PDF), and extended X-ray absorption fine structure (EXAFS) studies demonstrated that both dopants lowered oxygen activity by reducing Ni content, thereby inhibiting the redox reactions of Ni and Mn at high voltages [149]. Ion doping effectively suppressed the J–T effect and lattice oxygen release, reducing surface cracks and limiting the migration and dissolution of TM ions. This ion modification strategy also preserved the integrity of the CEI under high-voltage conditions, thereby minimizing interfacial side reactions.

### 4.3. Electrolyte Optimization

The electrolyte composition plays a critical role in determining the formation and stability of the CEI during cycling. Under high voltages, the instability of the CEI can lead to cathode surface corrosion, surface reconstruction, and dissolution of TM ions, all of which degrade the performance of SIBs. To enhance the structural integrity of the CEI, strategies such as adding electrolyte additives and designing low-solvation structures have been verified as being effective.

In recent years, many electrolyte additives have been explored to stabilize the electrode–electrolyte interface, including methyl ethyl carbonate (EMC), vinyl fluoro-carbonate (FEC), vinylene carbonate (VC), etc. In organic electrolytes, the CEI typically comprises various organic compounds formed via redox reactions and the catalytic decomposition of electrolyte solvents. This CEI is often unstable, resulting in continuous electrolyte degradation, surface reconstruction, and resulting rapid capacity decay [150]. Chou’s team introduced tri (pentafluorophenyl) borane (TPFPB) as an electrolyte additive to optimize the composition of the electrolyte, which tends to form an NaF-rich CEI. Their work reveals that the TPFPB additive could improve high-voltage performance and promote the formation of a uniform, compact CEI layer (7.4~10.4 nm) on the cathode surface. This method effectively reduces excessive electrolyte oxidation at elevated temperatures and minimizes the accumulation of side reaction products [151].

**Figure 10:**
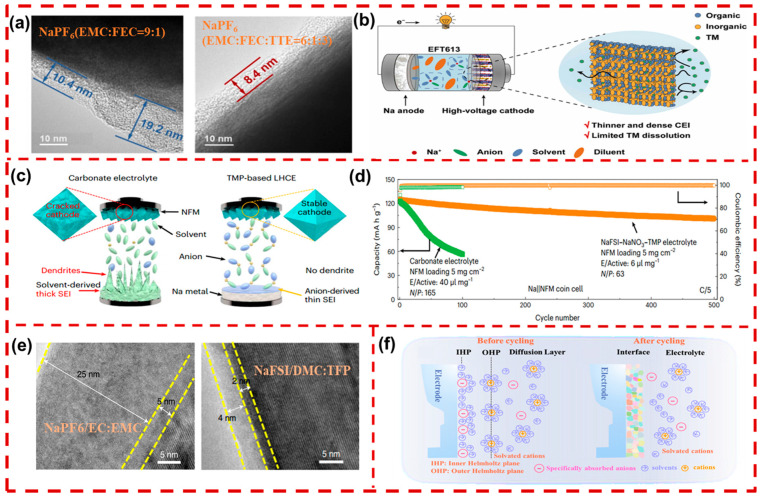
(**a**) TEM image of the Na-LMNM’T cathode in the NaPF_6_ (EMC/FEC) in a 9:1 (EF91) and NaPF_6_ (EMC/FEC/TTE) in a 6:1:3 (EFT613) electrolyte. (**b**) Schematic illustrations of the solvation structure and the CEIs in the EFT613 electrolytes [152]. Copyright 2024, Elsevier. (**c**) Illustration of the components with a Na-metal battery after long-term cycling in the carbonate-based electrolyte (**left**) and NaFSI–NaNO_3_–TMP electrolyte (TMP-based LHCE, **right**). (**d**) Cycling performance of NFM with an active-material loading of 5mg cm^−2^ [155]. Copyright 2024, Springer Nature. (**e**) Cryo-TEM images of an NMC cathode after 100 cycles in NaPF_6_/EC: EMC and NaFSI/DMC: TFP electrolytes (yellow dashed lines indicate the interfaces of the CEI and the surface reconstruction region of NMC) [156]. Copyright 2022, Springer Nature. (**f**) Schematic illustration of the structure of the interface corresponding to before and after electrochemical cycling [157]. Copyright 2024, Wiley.

In traditional low-concentration (<1 M) ether-based electrolytes, the decomposition of free solvent molecules on the cathode surface leads to the formation of an unstable CEI. To mitigate this issue, increasing the salt concentration to create a high-concentration electrolyte (HCE) can promote the formation of a more stable CEI. However, the HCE often exhibits poor wettability with the cathode and increases the viscosity, which hinders fast Na^+^ transport. To overcome these limitations, Zhang et al. introduced an inert diluent bis(2,2,2-trifluoroethyl) ether (BTFE) to formulate a localized high-concentration electrolyte (LHCE). It was revealed that employing the LHCE demonstrated significantly lower electrolyte resistance and reduced CEI interface resistance compared to using a conventional HCE [152]. Similarly, Xin et al. developed a 1M NaPF_6_ EMC/FEC/1,1,2,2-Tetrafluoroethyl 2,2,3,3-tetrafluoropropylether (TTE) (6:1:3 vol ratio, EFT613) for a P2-Na_0.7_Li_0.03_Mg_0.03_Ni_0.27_Mn_0.6_Ti_0.07_O_2_ cathode. The TEM image (Figure 10a) revealed that EFT613 generates a more uniform CEI (~8.4 nm), which could significantly mitigate TM ion dissolution under high voltages and accelerate Na^+^ transport kinetics, thereby enhancing overall electrochemical performance. As shown in Figure 10b, the inclusion of TTE enhances the interaction between free EMC molecules and Na^+^, thereby improving the oxidative stability of the electrolyte [153]. Sun et al. explored succinic anhydride (SA) and FEC as electrolyte additives in a propylene carbonate (PC)-based electrolyte, resulting in a complete and uniform CEI on the Na_0.6_Li_0.15_Ni_0.15_Mn_0.55_Cu_0.15_O_2_ (NLNMC) surface. Furthermore, their results indicated that the addition of SA reduced total CO_2_ generation by 17.0% during the initial cycle and 56.6% during the second cycle, demonstrating its effectiveness in suppressing electrolyte decomposition. Concurrently, the decomposition of FEC forms a NaF-rich CEI, which facilitates Na^+^ diffusion [154]. Manthiram and his colleagues explored NaNO_3_ as a diluent in a sodium bis(fluorosulfonyl)imide (NaFSI) electrolyte, complemented by a non-flammable trimethyl phosphate (TMP) solvent. As shown in Figure 10c, the PC-based electrolytes with free solvent molecules tend to undergo continuous reactions with the Na(Ni_0.3_Fe_0.4_Mn_0.3_)O_2_ (NNFM) cathode, leading to the significant generation of cracks. Conversely, the NaFSI-NaNO_3_-TMP electrolyte effectively displaces TMP solvent molecules within the primary solvation shell of Na^+^, which is driven by the synergistic interaction between NaFSI and NaNO_3_, facilitating the formation of a more stable CEI. As illustrated in Figure 10d, the NNFM utilizing the NaFSI-NaNO_3_-TMP electrolyte achieves both higher initial capacity and superior cycling stability than that of conventional carbonate-based electrolytes [155].

Na^+^ transfer in electrolytes and electrodes experiences solvation and desolvation processes during charging/discharging. The solvated complexes formed after Na^+^ solvation are relatively large, leading to a desolvation process that often exhibits a high energy barrier. Without a sufficient external driving force, achieving effective desolvation on the surface of Na_x_TMO_2_ is still a challenge. Zhang et al. selected low-solvation electrolytes to optimize the solvation structure, thereby enabling the formation of a stable CEI on NaNi_0.68_Mn_0.22_Co_0.1_O_2_ (NMC). They developed a NaFSI/dimethyl carbonate (DMC): triethyl phosphate (TFP) electrolyte by employing a low-polarity TFP solvent to replace traditional high-polarity carbonate solvents. As shown in Figure 10e, the NMC cathode using the NaFSI/DMC: TEP electrolyte exhibited a CEI with a thickness of only 4 nm, indicating that it effectively suppressed interfacial side reactions. Additionally, the TFP solvent significantly mitigates surface reconstruction, resulting in a phase transition layer of only 2 nm, while the conventional NaPF_6_/EC: EMC electrolyte produced a thicker 5 nm layer [156].

Wang et al. discovered that a double-layer structure known as the Helmholtz plane (HP) forms due to dielectric disparities between the Na_x_TMO_2_ and electrolyte. As illustrated in Figure 10f, the innermost layer, referred to as the inner Helmholtz plane (IHP), could adsorb anions and small-to-medium solvent molecules via electrostatic interactions, thereby establishing the initial interface. The outer Helmholtz plane (OHP), positioned further from the cathode surface, undergoes desolvation under an applied electric field. The decomposed solvent molecules and anions continuously contribute to the subsequent growth of the interfacial layer. This study indicates that modulating the IHP can dynamically stabilize the CEI, which is crucial for enhancing the long-term cycling stability of Na_x_TMO_2_. To optimize this effect, they designed an IHP structure dominated by PF_6_^−^, which effectively displaces diglyme (Dig) from the IHP, facilitating the rapid formation of a stable CEI. Compared to PC-based electrolytes, which exhibited higher NaF content on the CEI, the Dig-based electrolyte resulted in a lower NaF concentration after cycling. This finding highlights the effective suppression of NaPF_6_ decomposition in the Dig-based electrolyte, thus improving the stability of the CEI [157].

In recent years, the modification of the cathode solid-state electrolyte (SSE) interface has garnered significant attention. Compared to conventional organic liquid electrolytes, inorganic solid-state electrolytes (ISSEs) offer advantages such as no leakage, enhanced thermal stability, and low flammability, which improve safety. However, the microscopic, point-to-point physical contact between the cathode and solid electrolyte leads to increased interface impedance and sluggish Na^+^ migration kinetics [158,159]. To optimize these interface dynamics, composite cathodes consisting of cathode materials and electrolytes have been explored. For example, Wu et al. developed a composite cathode composed of NaCrO_2_ and Na_3-x_Y_1-x_Zr_x_Cl_6_ (NYZC) and revealed that NYZC significantly enhanced the stability and conductivity of the cathode, maintaining 89.3% of its capacity after over 1000 cycles at 1C [160]. In an effort to further improve cathode–SSE interface compatibility, Sun et al. designed a dual-anion chloroxide sublattice solid-state electrolyte, Na_2_O_2_-MCl_y_ (NMOC; M = Hf, Zr, Ta; y = 4, 5). XPS and X-ray absorption fine structure (XAFS) analysis confirmed that the NMOC electrolyte demonstrated excellent electrochemical stability when paired with the Na_0.85_Mn_0.5_Ni_0.4_Fe_0.1_O_2_ cathode. Additionally, the appropriate Young’s modulus of the NMOC electrolyte exhibited excellent structural durability, effectively compensating for volume expansion during the Na^+^ insertion process at the cathode [161].

The aforementioned analysis indicates that the optimization of electrolyte strategies plays a crucial role in the development of a high-quality CEI. A uniform and dense CEI not only mitigates the dissolution of TM ions in Na_x_TMO_2_ but also suppresses the generation of reactive oxygen species under elevated voltages, thereby facilitating the advancement of long-life SIBs.

## 5. Summary and Outlook

This review outlines the interfacial degradation mechanisms of Na_x_TMO_2_ cathodes, including air instability, phase transitions, TM ion migration, and interfacial side reactions. At the stage of material storage, Na_x_TMO_2_ is prone to reacting with atmospheric components, resulting in surface structure degradation. In high-voltage charging/discharging, extensive Na^+^ extraction induces TMO_2_ slippage and increases repulsion between O-O bonds, thereby accelerating lattice oxygen release and TM ion migration, which ultimately compromise battery longevity. Furthermore, irreversible phase transitions lead to the formation of disordered spinel and rock-salt phases during prolonged cycling, which hinders Na^+^ transport. The stresses associated with these phase transitions can also generate intergranular cracks, allowing electrolyte infiltration into the Na_x_TMO_2_ structure. Additionally, the continually exposed surface exacerbates parasitic reactions between the electrolyte and Na_x_TMO_2_. These parasitic reactions not only degrade the cathode’s surface structure but also contribute to the dissolution of TM ions, further impacting cycling stability.

**Figure 11:**
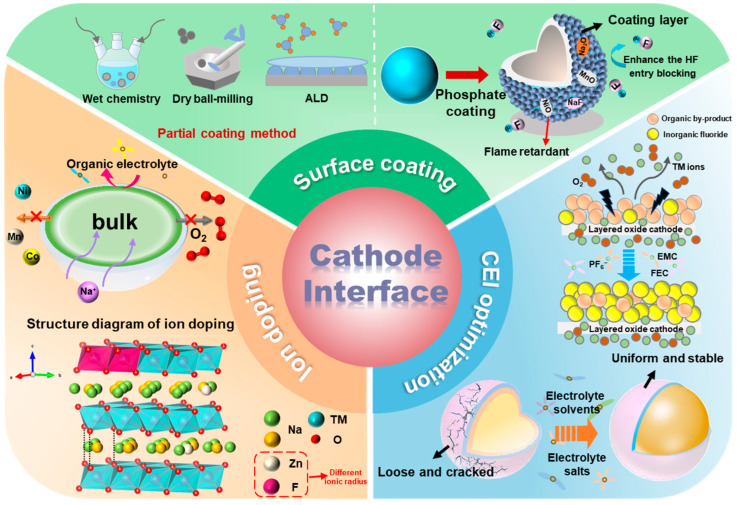
Optimization strategy of interface issues of the Na_x_TMO_2_ cathode.

Meanwhile, as illustrated in Figure 11, various interface modification strategies have been listed, including surface engineering, ion doping, and electrolyte optimization. Surface engineering could significantly stabilize the structure of cathode materials and mitigate phase transformation stress, thereby improving the cycling stability of Na_x_TMO_2_ cathodes. The coating layer could serve as a physical barrier to inhibit the dissolution of TM ions, prevent interfacial side reactions, and remove residual surface alkali during synthesis. For certain coatings (e.g., NaPO_3_), they can also eliminate HF and H_2_O generated by the electrolyte, preventing chemical corrosion of the electrolyte. Some solid-state electrolyte coatings could offer specific ion channels, facilitating Na^+^ transport kinetics. Ion doping could stabilize the lattice structure, suppress phase transformations, and alleviate stress-induced surface cracking of Na_x_TMO_2_. Furthermore, it could inhibit the migration and dissolution of TM ions, reducing surface reconstruction and thus improving the cycling stability and rate capability. Electrolyte optimization is an effective strategy to regulate the structure and stability of the CEI. An inorganic-rich CEI (NaF-rich) could crucially reduce the side reactions between the Na_x_TMO_2_ and electrolyte. Moreover, adjusting the IHP can dynamically stabilize the CEI, which is also essential for improving the long-term cycling stability of Na_x_TMO_2_. As shown in Table 1, a comparison of the electrochemical performance of modified Na_x_TMO_2_-based cathode materials is presented.

Although numerous modification strategies have been explored for enhancing the interface stability of Na_x_TMO_2_ cathodes, conventional techniques still impose limitations. One critical aspect is to ensure that the coating materials are compatible with the Na_x_TMO_2_ cathode. Factors such as particle morphology and physicochemical properties are worth taking into account. While thin coatings promote Na^+^ transport, achieving uniform modification layers often requires complex techniques such as hydrothermal or solvothermal processes, which are not feasible for large-scale applications. Future research in surface engineering of Na_x_TMO_2_ cathodes should focus on precise, scalable methods such as ALD, pulsed laser deposition (PLD), or chemical vapor deposition (CVD) to ensure cost-effectiveness and uniformity. Additionally, surface doping could potentially boost the structural and chemical stability of the Na_x_TMO_2_ cathode. Unlike bulk doping, surface doping concentrates dopants on the outermost layers, achieving enhanced surface stability with a reduced amount of dopants. This approach helps minimize initial capacity loss to achieve higher energy density. Identifying electrolytes that are compatible with high-voltage Na_x_TMO_2_ cathodes is also worthy of attention. Solving the above problems contributes to the development of long-life and high-rate-capability Na_x_TMO_2_ cathodes for SIBs.

Advanced characterization techniques are pivotal for investigating the interface stability of these Na_x_TMO_2_ cathodes. For example, STEM is instrumental for visualizing the phase structures on cathode surfaces, while EELS is employed to ascertain the valence states of TM ions from the surface to the core. Furthermore, in situ TEM could offer real-time observation of interface evolution during charging/discharging cycles. TOF-SIMS could provide a detailed analysis of the CEI’s thickness and composition through etching techniques. Despite the potential of Na_x_TMO_2_ cathodes, a gap in mechanism research has hindered their full acceptance for industrial production.

Overall, with strategic surface engineering and material optimization, an interfacial stable Na_x_TMO_2_ cathode can achieve superior electrochemical performance, meeting the cost-effective requirements of SIBs in future energy storage systems. It is hoped that this work could guide the design of interfacial stable Na_x_TMO_2_ cathodes for SIBs.

## Figures and Tables

**Table 1:** The electrochemical performance comparison of Na_x_TMO_2._

Cathode Materials	Modified Methods	Voltage (V)	Reversible Capacity (mA h g^−1^)	Capacity Retention	Ref.
P2-Na_2/3_[Ni_1/3_Mn_2/3_]O_2_	Surface coating	2.5–4.3	192 at 0.5 C	88.4% after 50 cycles	[114]
P2-Na_2/3_Ni_1/3_Mn_2/3_O_2_	Surface coating	1.5–4.8	150 at 0.5 C	61% after 100 cycles	[115]
P3/P2-Na_2/3_Ni_1/3_Mn_2/3_O_2_	Surface coating	1.5–4.1	105 at 5 C	87% after 300 cycles	[116]
O3-NaMn_1/3_Fe_1/3_Ni_1/3_O_2_	Surface coating	1.5–4.6	128 at 1 C	77.7% after 100 cycles	[121]
P2-Na_2/3_[Ni_1/3_Mn_2/3_]O_2_	Surface coating	2.5–4.3	121 at 0.5 C	75.4% after 200 cycles	[122]
O3-Na[Ni_0.5_Mn_0.5_]O_2_	Surface coating	2.0–4.2	187 at 0.1 C	75% after 100 cycles	[123]
P2-Na_0.85_Li_0.12_Ni_0.22_Mn_0.66_O_2_	Surface coating	2.0–4.3	111.4 at 1 C	94.5% after 100 cycles	[124]
P2-Na_0.6_[Li_0.2_Mn_0.8_]O_2_	Surface coating	2.0–4.5	103 at 1 C	85.4% after 40 cycles	[125]
P3-Na_2/3_Ni_1/2_Mn_1/2_O_2_	Surface coating	2.0–4.3	210 at 0.5 C	90% after 20 cycles	[128]
P2-Na_2/3_[Ni_1/3_Mn_2/3_]O_2_	Surface coating	1.5–4.3	191 at 0.1 C	80% after 50 cycles	[129]
P2-Na_2/3_[Ni_1/3_Mn_2/3_]O_2_	Surface coating	2.5–4.3	125 at 1 C	74% after 200 cycles	[131]
O3-NaNi_0.4_Fe_0.2_Mn_0.4_O_2_	Surface coating	2.0–4.0	147.6 at 0.1 C	74% after 100 cycles	[132]
P2-Na_2/3_[Ni_1/3_Mn_2/3_]O_2_	Surface coating	2.5–4.3	120.8 at 5 C	63.8% after 300 cycles	[133]
P2-Na_0.65_[Mn_0.70_Ni_0.16_Co_0.14_]O_2_	Surface coating	1.5–4.3	166.7 at 1 C	88.3% after 100 cycles	[134]
O3-Na[Ni_0.65_Co_0.08_Mn_0.27_]O_2_	Surface coating	1.5–4.1	168 at 75 mA g^−1^	90% after 50 cycles	[135]
O3-NaNi_1/3_Fe_1/3_Mn_1/3_O_2_	Surface coating	2.0–4.3	135 at 2 C	70% after 300 cycles	[136]
P2-Na_0.67_Ni_0.33_Mn_0.67_O_2_	Element doping	2.0–4.5	125 at 0.1 C	81.7% after 100 cycles	[140]
P2-Na_0.67_Ni_0.33_Mn_0.37_Ti_0.3_O_2_	Element doping	2.0–4.3	92 at 1 C	78% after 200 cycles	[141]
P2-Na_0.7_Fe_0.1_Mn_0.75_□_0.15_O_2_	Element doping	2.0–4.5	115 at 100 mA g^−1^	70.1% after 200 cycles	[142]
P2-Na_0.78_Ni_0.23_□_0.08_Mn_0.69_O_2_	Element doping	2.0–4.3	153.9 at 0.1 C	87.1% after 50 cycles	[143]
P2–Na_0.66_Li_0.18_Fe_0.12_Mn_0.7_O_2_	Element doping	1.5–4.5	190 at 0.1 C	87% after 80 cycles	[144]
P2-Na_0.67_Li_0.1_Fe_0.37_Mn_0.53_O_2_	Element doping	2.0–4.5	105 at 1 C	84.5% after 100 cycles	[145]
P2-Na_7/9_[Li_1/9_Ni_1/9_Mg_1/9_Mn_6/9_]O_2_	Element doping	1.5–4.5	155 at 100 mA g^−1^	90.3% after 100 cycles	[146]
P2-Na_0.8_Li_0.24_Al_0.03_Mn_0.73_O_2_	Element doping	2.0–4.5	264.3 at 0.2 C	66.2% after 50 cycles	[147]
O3-Na_0.9_Ni_0.32_Zn_0.08_Fe_0.1_Mn_0.3_Ti_0.2_O_2_	Element doping	2.0–4.0	120 at 200 mA g^−1^	84.2% after 800 cycles	[148]
P2-Na_0.67_Mg_0.1_Ni_0.23_Mn_0.67_O_2_	Element doping	2.5–4.4	118 at 20 mA g^−1^	49.1% after 500 cycles	[149]
P2-Na_0.7_Li_0.03_Mg_0.03_Ni_0.2_7Mn_0.6_Ti_0.07_O_2_	Electrolyte optimization	2.5–4.3	129 at 1 C	87.3% after 200 cycles	[153]
P2-Na/Na_0.6_Li_0.15_Ni_0.15_Mn_0.55_Cu_0.15_O_2_	Electrolyte optimization	2.0–4.5	110.6 at 0.2 C	99.4% after 100 cycles	[154]
O3-Na[Ni_0.3_Fe_0.4_Mn_0.3_]O_2_	Electrolyte optimization	2.0–4.0	126 at 1/5 C	80% after 500 cycles	[155]
P2-Na_0.67_Fe_0.1_Mn_0.9_O_2_	Electrolyte optimization	1.5–4.3	140 at 200 mA g^−1^	90.4% after 200 cycles	[157]
Na_0.85_Mn_0.5_Ni_0.4_Fe_0.1_O_2_	Electrolyte optimization	2.0–4.0	111 at 0.2 C	78% after 700 cycles	[161]

## Data Availability

The data that support the findings of this study are available from the corresponding author upon reasonable request.

## References

[1] Ma W., Wan S., Cui X., Hou G., Xiao Y., Rong J., Chen S. (2023). Exploration and Application of Self-Healing Strategies in Lithium Batteries. Adv. Funct. Mater..

[2] Yang T., Xu X., Yang Y., Fan W., Wu Y., Ji S., Zhao J., Liu J., Huo Y. (2024). Recent Advances and Perspectives of Covalent Organic Frameworks for Alkali-Ion Batteries. Energy Mater. Adv..

[3] Masias A., Marcicki J., Paxton W.A. (2021). Opportunities and Challenges of Lithium Ion Batteries in Automotive Applications. ACS Energy Lett..

[4] Peng C., Xu X., Li F., Xi L., Zeng J., Song X., Wan X., Zhao J., Liu J. (2023). Recent progress of promising cathode candidates for sodium-ion batteries: Current issues, strategy, challenge, and prospects. Small Struct..

[5] Wang Y., Xu X., Wu Y., Li F., Fan W., Wu Y., Ji S., Zhao J., Liu J., Huo Y. (2024). Facile Galvanic Replacement Construction of Bi@C Nanosheets Array as Binder-Free Anodes for Superior Sodium-Ion Batteries. Adv. Energy Mater..

[6] Wang J., Zhu Y.-F., Su Y., Guo J.-X., Chen S., Liu H.-K., Dou S.-X., Chou S.-L., Xiao Y. (2024). Routes to high-performance layered oxide cathodes for sodium-ion batteries. Chem. Soc. Rev..

[7] Wang W., Gang Y., Hu Z., Yan Z., Li W., Li Y., Gu Q.-F., Wang Z., Chou S.-L., Liu H.-K. (2020). Reversible structural evolution of sodium-rich rhombohedral Prussian blue for sodium-ion batteries. Nat. Commun..

[8] Li Z., Li F., Xu X., Zeng J., Zhang H., Xi L., Wu Y., Zhao L., Chen J., Liu J. (2024). A scalable approach to Na_4_Fe_3_(PO_4_)_2_P_2_O_7_@carbon/expanded graphite as cathode for ultralong-lifespan and low-temperature sodium-ion batteries. Chin. Chem. Lett..

[9] Liu X., Yuan C., Zheng X., Cheng G., Qian H., Zheng B., Lu X., Yang Y., Zhu Y., Xiang Y. (2024). Stabilizing Interlayer Repulsion in Layered Sodium-Ion Oxide Cathodes via Hierarchical Layer Modification. Adv. Mater..

[10] Liu Z., Xu X., Ji S., Zeng L., Zhang D., Liu J. (2020). Recent Progress of P2-Type Layered Transition-Metal Oxide Cathodes for Sodium-Ion Batteries. Chem. Eur. J..

[11] Yabuuchi N., Kajiyama M., Iwatate J., Nishikawa H., Hitomi S., Okuyama R., Usui R., Yamada Y., Komaba S. (2012). P2-type Na_x_[Fe_1/2_Mn_1/2_]O_2_ made from earth-abundant elements for rechargeable Na batteries. Nat. Mater..

[12] Jamil S., Li C., Fasehullah M., Liu P., Xiao F., Wang H., Bao S., Xu M. (2022). Ni/Li antisite induced disordered passivation layer for high-Ni layered oxide cathode material. Energy Storage Mater..

[13] Li Y., Zhou Q., Weng S., Ding F., Qi X., Lu J., Li Y., Zhang X., Rong X., Lu Y. (2022). Interfacial engineering to achieve an energy density of over 200 Wh kg^−1^ in sodium batteries. Nat. Energy.

[14] Li H., Wang J., Xu S., Chen A., Lu H., Jin Y., Guo S., Zhu J. (2024). Universal Design Strategy for Air-Stable Layered Na-Ion Cathodes toward Sustainable Energy Storage. Adv. Mater..

[15] Wang P.-F., Weng M., Xiao Y., Hu Z., Li Q., Li M., Wang Y.-D., Chen X., Yang X., Wen Y. (2019). An Ordered Ni_6_-Ring Superstructure Enables a Highly Stable Sodium Oxide Cathode. Adv. Mater..

[16] Liu Z., Liu R., Xu S., Tian J., Li J., Li H., Yu T., Chu S., D’Angelo A.M., Pang W.K. (2024). Achieving a Deeply Desodiated Stabilized Cathode Material by the High Entropy Strategy for Sodium-ion Batteries. Angew. Chem. Int. Ed..

[17] Mariyappan S., Marchandier T., Rabuel F., Iadecola A., Rousse G., Morozov A.V., Abakumov A.M., Tarascon J.-M. (2020). The Role of Divalent (Zn^2+^/Mg^2+^/Cu^2+^) Substituents in Achieving Full Capacity of Sodium Layered Oxides for Na-Ion Battery Applications. Chem. Mater..

[18] Ren H., Li Y., Ni Q., Bai Y., Zhao H., Wu C. (2022). Unraveling Anionic Redox for Sodium Layered Oxide Cathodes: Breakthroughs and Perspectives. Adv. Mater..

[19] Zhang R., Wang C., Zou P., Lin R., Ma L., Yin L., Li T., Xu W., Jia H., Li Q. (2022). Compositionally complex doping for zero-strain zero-cobalt layered cathodes. Nature.

[20] You Y., Xin S., Asl H.Y., Li W., Wang P.-F., Guo Y.-G., Manthiram A. (2018). Insights into the Improved High-Voltage Performance of Li-Incorporated Layered Oxide Cathodes for Sodium-Ion Batteries. Chem.

[21] Piao Z., Ren H.-R., Lu G., Jia K., Tan J., Wu X., Zhuang Z., Han Z., Li C., Gao R. (2023). Stable Operation of Lithium Metal Batteries with Aggressive Cathode Chemistries at 4.9 V. Angew. Chem. Int. Ed..

[22] Lei Z.-Q., Guo Y.-J., Wang E.-H., He W.-H., Zhang Y.-Y., Xin S., Yin Y.-X., Guo Y.-G. (2022). Layered Oxide Cathode-Electrolyte Interface towards Na-Ion Batteries: Advances and Perspectives. Chem. Asian J..

[23] Kim D., Kang S.-H., Slater M., Rood S., Vaughey J.T., Karan N., Balasubramanian M., Johnson C.S. (2011). Enabling Sodium Batteries Using Lithium-Substituted Sodium Layered Transition Metal Oxide Cathodes. Adv. Energy Mater..

[24] An S., Karger L., Dreyer S.L., Hu Y., Barbosa E., Zhang R., Lin J., Fichtner M., Kondrakov A., Janek J. (2024). Improving cycling performance of the NaNiO_2_ cathode in sodium-ion batteries by titanium substitution. Mater. Futur..

[25] Jiang C., Wang Y., Xin Y., Zhou Q., Pang Y., Chen B., Wang Z., Gao H. (2024). A high-rate and air-stable cathode material for sodium-ion batteries: Yttrium-substituted O3-type Ni/Fe/Mn-based layered oxides. J. Mater. Chem. A.

[26] Lei Y., Ni J., Hu Z., Wang Z., Gui F., Li B., Ming P., Zhang C., Elias Y., Aurbach D. (2020). Surface Modification of Li-Rich Mn-Based Layered Oxide Cathodes: Challenges, Materials, Methods, and Characterization. Adv. Energy Mater..

[27] Alvarado J., Ma C., Wang S., Nguyen K., Kodur M., Meng Y.S. (2017). Improvement of the Cathode Electrolyte Interphase on P2-Na_2/3_Ni_1/_3Mn_2/3_O_2_ by Atomic Layer Deposition. ACS Appl. Mater. Interfaces.

[28] Xiao X., Lu P., Ahn D. (2011). Ultrathin Multifunctional Oxide Coatings for Lithium Ion Batteries. Adv. Mater..

[29] Sun Y., Wang C., Huang W., Zhao G., Duan L., Liu Q., Wang S., Fraser A., Guo H., Sun X. (2023). One-Step Calcination Synthesis of Bulk-Doped Surface-Modified Ni-Rich Cathodes with Superlattice for Long-Cycling Li-Ion Batteries. Angew. Chem..

[30] Liu Y., Fang X., Ge M., Rong J., Shen C., Zhang A., Enaya H.A., Zhou C. (2015). SnO_2_ coated carbon cloth with surface modification as Na-ion battery anode. Nano Energy.

[31] Chu Y., Mu Y., Zou L., Hu Y., Cheng J., Wu B., Han M., Xi S., Zhang Q., Zeng L. (2023). Thermodynamically Stable Dual-Modified LiF&FeF_3_ layer Empowering Ni-Rich Cathodes with Superior Cyclabilities. Adv. Mater..

[32] Li N., Ren J., Dang R., Wu K., Lee Y.L., Hu Z., Xiao X. (2019). Suppressing phase transition and improving electrochemical performances of O3-NaNi_1/3_Mn_1/3_Fe_1/3_O_2_ through ionic conductive Na_2_SiO_3_ coating. J. Power Sources.

[33] Deng J., Luo W.-B., Lu X., Yao Q., Wang Z., Liu H.-K., Zhou H., Dou S.-X. (2018). High Energy Density Sodium-Ion Battery with Industrially Feasible and Air-Stable O3-Type Layered Oxide Cathode. Adv. Energy Mater..

[34] Cheng C., Ding M., Yan T., Jiang J., Mao J., Feng X., Chan T.-S., Li N., Zhang L. (2022). Anionic Redox Activities Boosted by Aluminum Doping in Layered Sodium-Ion Battery Electrode. Small Methods.

[35] Wei S.-B., He Y.-J., Tang Y., Fu H.-W., Zhou J., Liang S.-Q., Cao X.-X. (2024). A Ca-substituted air-stable layered oxide cathode material with facilitated phase transitions for high-performance Na-ion batteries. Rare Met..

[36] Hwang J.-Y., Oh S.-M., Myung S.-T., Chung K.Y., Belharouak I., Sun Y.-K. (2015). Radially aligned hierarchical columnar structure as a cathode material for high energy density sodium-ion batteries. Nat. Commun..

[37] Cui Z., Li X., Bai X., Ren X., Ou X. (2023). A comprehensive review of foreign-ion doping and recent achievements for nickel-rich cathode materials. Energy Storage Mater..

[38] Liao Y., Yuan L., Han Y., Liang C., Li Z., Li Z., Luo W., Wang D., Huang Y. (2024). Pentafluoro(phenoxy)cyclotriphosphazene Stabilizes Electrode/Electrolyte Interfaces for Sodium-Ion. Pouch Cells of 145 Wh Kg^−1^. Adv. Mater..

[39] Liu Y.-F., Han K., Peng D.-N., Kong L.-Y., Su Y., Li H.-W., Hu H.-Y., Li J.-Y., Wang H.-R., Fu Z.-Q. (2023). Layered oxide cathodes for sodium-ion batteries: From air stability, interface chemistry to phase transition. InfoMat.

[40] Yu L., Chang Y.-X., Liu M., Feng Y.-H., Si D., Zhu X., Wang X.-Z., Wang P.-F., Xu S. (2023). O3-Type Na_0.95_Ni_0.40_Fe_0.15_Mn_0.3_Ti_0.15_O_2_ Cathode Materials with Enhanced Storage Stability for High-Energy Na-Ion Batteries. ACS Appl. Mater. Interfaces.

[41] Zhao S., Ning F., Yu X., Guo B., Teófilo R.F., Huang J., Shi Q., Wu S., Feng W., Zhao Y. (2024). Inhomogeneous Coordination in High-Entropy O3-Type Cathodes Enables Suppressed Slab Gliding and Durable Sodium Storage. Angew. Chem. Int. Ed..

[42] Wang X.-Z., Zuo Y., Qin Y., Zhu X., Xu S.-W., Guo Y.-J., Yan T., Zhang L., Gao Z., Yu L. (2024). Fast Na^+^ Kinetics and Suppressed Voltage Hysteresis Enabled by a High-Entropy Strategy for Sodium Oxide Cathodes. Adv. Mater..

[43] Wang Q.-C., Shadike Z., Li X.-L., Bao J., Qiu Q.-Q., Hu E., Bak S.-M., Xiao X., Ma L., Wu X.-J. (2021). Tuning Sodium Occupancy Sites in P2-Layered Cathode Material for Enhancing Electrochemical Performance. Adv. Energy Mater..

[44] Cheng Z., Zhao B., Guo Y.-J., Yu L., Yuan B., Hua W., Yin Y.-X., Xu S., Xiao B., Han X. (2022). Mitigating the Large-Volume Phase Transition of P2-Type Cathodes by Synergetic Effect of Multiple Ions for Improved Sodium-Ion Batteries. Adv. Energy Mater..

[45] Guo S., Li Q., Liu P., Chen M., Zhou H. (2017). Environmentally stable interface of layered oxide cathodes for sodium-ion batteries. Nat. Commun..

[46] Zuo W., Qiu J., Liu X., Ren F., Liu H., He H., Luo C., Li J., Ortiz G.F., Duan H. (2020). The stability of P2-layered sodium transition metal oxides in ambient atmospheres. Nat. Commun..

[47] Zuo W., Xiao Z., Zarrabeitia M., Xue X., Yang Y., Passerini S. (2022). Guidelines for Air-Stable Lithium/Sodium Layered Oxide Cathodes. ACS Mater. Lett..

[48] Zhang R., Yang S., Li H., Zhai T., Li H. (2022). Air sensitivity of electrode materials in Li/Na ion batteries: Issues and strategies. InfoMat.

[49] You Y., Dolocan A., Li W., Manthiram A. (2019). Understanding the Air-Exposure Degradation Chemistry at a Nanoscale of Layered Oxide Cathodes for Sodium-Ion Batteries. Nano Lett..

[50] Duffort V., Talaie E., Black R., Nazar L.F. (2015). Uptake of CO_2_ in Layered P2-Na_0.67_Mn_0.5_Fe_0.5_O_2_: Insertion of Carbonate Anions. Chem. Mater..

[51] Yang Y., Wang Z., Du C., Wang B., Li X., Wu S., Li X., Zhang X., Wang X., Niu Y. (2024). Decoupling the air sensitivity of Na-layered oxides. Science.

[52] Lu Z., Dahn J.R. (2001). Intercalation of Water in P2, T2 and O2 Structure A_z_[Co_x_Ni_1/3-x_Mn_2/3_]O_2_. Chem. Mater.

[53] Huang Y., Zhao L., Li L., Xie M., Wu F., Chen R. (2019). Electrolytes and Electrolyte/Electrode Interfaces in Sodium-Ion Batteries: From Scientific Research to Practical Application. Adv. Mater..

[54] Torres R.M., Manthiram A. (2024). Delineating the Effects of Transition-Metal-Ion Dissolution on Silicon Anodes in Lithium-Ion Batteries. Small.

[55] Chu S., Guo S. (2024). From Rotten to Magical: Transition Metal Migration in Layered Sodium-Ion Battery Cathodes. Adv. Funct. Mater..

[56] Xu X., Hu S., Pan Q., Huang Y., Zhang J., Chen Y., Wang H., Zheng F., Li Q. (2024). Enhancing Structure Stability by Mg/Cr Co-Doped for High-Voltage Sodium-Ion Batteries. Small.

[57] Maiti S., Konar R., Sclar H., Grinblat J., Talianker M., Tkachev M., Wu X., Kondrakov A., Nessim G.D., Aurbach D. (2022). Stabilizing High-Voltage Lithium-Ion Battery Cathodes Using Functional Coatings of 2D Tungsten Diselenide. ACS Energy Lett..

[58] Wang Y., Zhao X., Jin J., Shen Q., Zhang N., Qu X., Liu Y., Jiao L. (2022). Low-cost layered oxide cathode involving cationic and anionic redox with a complete solid-solution sodium-storage behavior. Energy Storage Mater..

[59] Xiao Z., Xia F., Xu L., Wang X., Meng J., Wang H., Zhang X., Geng L., Wu J., Mai L. (2022). Suppressing the Jahn–Teller Effect in Mn-Based Layered Oxide Cathode toward Long-Life Potassium-Ion. Batteries. Adv. Funct. Mater..

[60] Banerjee A., Shilina Y., Ziv B., Ziegelbauer J.M., Luski S., Aurbach D., Halalay I.C. (2017). On the Oxidation State of Manganese Ions in Li-Ion Battery Electrolyte Solutions. J. Am. Chem. Soc..

[61] Zhang Y., Zheng S., Meng C., Liu H., Dong C., Shi X., Das P., Huang R., Yu Y., Wu Z.-S. (2023). A Near-Surface Structure Reconfiguration Strategy to Regulate Mn^3+^/Mn^4+^ and O^2−^/(O_2_)^n−^ Redox for Stabilizing Lithium-Rich Oxide Cathode. Adv. Funct. Mater..

[62] Asl H.Y., Manthiram A. (2020). Reining in dissolved transition-metal ions. Science.

[63] Zuo C., Hu Z., Qi R., Liu J., Li Z., Lu J., Dong C., Yang K., Huang W., Chen C. (2020). Double the Capacity of Manganese Spinel for Lithium-Ion Storage by Suppression of Cooperative Jahn-Teller Distortion. Adv. Energy Mater..

[64] Gao S., Zhu Z., Fang H., Feng K., Zhong J., Hou M., Guo Y., Li F., Zhang W., Ma Z. (2024). Regulation of Coordination Chemistry for Ultrastable Layered Oxide Cathode Materials of Sodium-Ion Batteries. Adv. Mater..

[65] Chen Z., Yang M., Chen G., Tang G., Huang Z., Chu M., Qi R., Li S., Wang R., Wang C. (2022). Triggering anionic redox activity in Fe/Mn-based layered oxide for high-performance sodium-ion batteries. Nano Energy.

[66] Li Y., Gao Y., Wang X., Shen X., Kong Q., Yu R., Lu G., Wang Z., Chen L. (2018). Iron migration and oxygen oxidation during sodium extraction from NaFeO_2_. Nano Energy.

[67] Gao X., Fang L., Wang H., Lee S., Liu H., Zhang S., Gao J., Mei Y., Park M., Zhang J. (2023). Origin of Fast Capacity Decay in Fe-Mn Based Sodium Layered Oxides. Adv. Funct. Mater..

[68] Lee E., Brown D.E., Alp E.E., Ren Y., Lu J., Woo J.-J., Johnson C.S. (2015). New Insights into the Performance Degradation of Fe-Based Layered Oxides in Sodium-Ion Batteries: Instability of Fe^3+^/Fe^4+^ Redox in α-NaFeO_2_. Chem. Mater..

[69] Jiang M., Qian G., Liao X.-Z., Ren Z., Dong Q., Meng D., Cui G., Yuan S., Lee S.-J., Qin T. (2022). Revisiting the capacity-fading mechanism of P2-type sodium layered oxide cathode materials during high-voltage cycling. J. Energy Chem..

[70] Wang P.-F., Jin T., Zhang J., Wang Q.-C., Ji X., Cui C., Piao N., Liu S., Xu J., Yang X.-Q. (2020). Elucidation of the Jahn-Teller effect in a pair of sodium isomer. Nano Energy.

[71] Li N., Yin W., Wang B., Wang F., Xiao X., Zhao J., Zhao E. (2024). Lowering Sodium-Storage Lattice Strains of Layered Oxide Cathodes by Pushing Charge Transfer on Anions. Energy Environ. Mater..

[72] Xu G.-L., Liu X., Zhou X., Zhao C., Hwang I., Daali A., Yang Z., Ren Y., Sun C.-J., Chen Z. (2022). Native lattice strain induced structural earthquake in sodium layered oxide cathodes. Nat. Commun..

[73] Jin T., Wang P.-F., Wang Q.-C., Zhu K., Deng T., Zhang J., Zhang W., Yang X.-Q., Jiao L., Wang C. (2020). Realizing Complete Solid-Solution Reaction in High Sodium Content P2-Type Cathode for High-Performance Sodium-Ion Batteries. Angew. Chem. Int. Ed..

[74] Wang K., Yan P., Sui M. (2018). Phase transition induced cracking plaguing layered cathode for sodium-ion battery. Nano Energy.

[75] Wang C., Liu L., Zhao S., Liu Y., Yang Y., Yu H., Lee S., Lee G.-H., Kang Y.-M., Liu R. (2021). Tuning local chemistry of P2 layered-oxide cathode for high energy and long cycles of sodium-ion battery. Nat. Commun..

[76] Somerville J.W., Sobkowiak A., Tapia-Ruiz N., Billaud J., Lozano J.G., House R.A., Gallington L.C., Ericsson T., Häggström L., Roberts M.R. (2019). Nature of the “Z”-phase in layered Na-ion battery cathodes. Energy Environ. Sci..

[77] Zhao C., Yao Z., Wang Q., Li H., Wang J., Liu M., Ganapathy S., Lu Y., Cabana J., Li B. (2020). Revealing High Na-Content P2-Type Layered Oxides as Advanced Sodium-Ion Cathodes. J. Am. Chem. Soc..

[78] Park Y.J., Choi J.U., Jo J.H., Jo C.-H., Kim J., Myung S.-T. (2019). A New Strategy to Build a High-Performance P’2-Type Cathode Material through Titanium Doping for Sodium-Ion Batteries. Adv. Funct. Mater..

[79] Chen X., Zheng S., Liu P., Sun Z., Zhu K., Li H., Liu Y., Jiao L. (2023). Fluorine Substitution Promotes Air-Stability of P’2-Type Layered Cathodes for Sodium-Ion Batteries. Small.

[80] Y Zhao Y., Liu Q., Zhao X., Mu D., Tan G., Li L., Chen R., Wu F. (2023). Structure evolution of layered transition metal oxide cathode materials for Na-ion batteries: Issues, mechanism and strategies. Mater. Today.

[81] Kim J.C., Kwon D.-H., Yang J.H., Kim H., Bo S.-H., Wu L., Kim H., Seo D.-H., Shi T., Wang J. (2020). Direct Observation of Alternating Octahedral and Prismatic Sodium Layers in O3-Type Transition Metal Oxides. Adv. Energy Mater..

[82] Zhang K., Xu Z., Li G., Luo R.-J., Ma C., Wang Y., Zhou Y.-N., Xia Y. (2023). Regulating Phase Transition and Oxygen Redox to Achieve Stable High-Voltage O3-Type Cathode Materials for Sodium-Ion Batteries. Adv. Energy Mater..

[83] Cheng F., Xu J., Wei P., Cheng Z., Liao M., Sun S., Xu Y., Li Q., Fang C., Lin Y. (2023). Interface Engineering via Regulating Electrolyte for High-Voltage Layered Oxide Cathodes-Based Li-Ion Batteries. Adv. Sci..

[84] Zhao w., Wang M., Lin H., Kim K., He R., Feng S., Liu H. (2024). Research progress on electrolyte key salts for sodium-ion batteries. Prog. Nat. Sci. Mater. Int..

[85] Zou L., He Y., Liu Z., Jia H., Zhu J., Zheng J., Wang G., Li X., Xiao J., Liu J. (2020). Unlocking the passivation nature of the cathode–air interfacial reactions in lithium ion batteries. Nat. Commun..

[86] Yu X., Manthiram A. (2018). Electrode–electrolyte interfaces in lithium-based batteries. Energy Environ. Sci..

[87] Sun L., Zeng J., Wan X., Peng C., Wang J., Lin C., Zhu M., Liu J. (2024). Recent progress of interface modification of layered oxide cathode material for sodium-ion batteries. Electron.

[88] Wang E., Niu Y., Yin Y.-X., Guo Y.-G. (2021). Manipulating Electrode/Electrolyte Interphases of Sodium-Ion Batteries: Strategies and Perspectives. ACS Mater. Lett..

[89] Wen B., Deng Z., Tsai P.-C., Lebens-Higgins Z.W., Piper L.F.J., Ong S.P., Chiang Y.-M. (2020). Ultrafast ion transport at a cathode–electrolyte interface and its strong dependence on salt solvation. Nat. Energy.

[90] Wu L., Huo H., Zhou Q., Yin X., Ma Y., Wang J., Du C., Zuo P., Yin G., Gao Y. (2022). Developing a Double Protection Strategy for High-Performance Spinel LiNi_0.5_Mn_1.5_O_4_ Cathodes. ACS Appl. Energy Mater..

[91] Moeez I., Susanto D., Chang W., Lim H.-D., Chung K.Y. (2021). Artificial cathode electrolyte interphase by functional additives toward long-life sodium-ion batteries. Chem. Eng. J..

[92] Liu J., Pei B., Su Q., Ren W., Ou H., Liang S., Cao X. (2023). In situ construction of uniform and robust cathode-electrolyte interface toward high-stable P2-Type sodium layered oxide cathodes. J. Power Sources.

[93] Farahmandjou M., Zhao S., Lai W.-H., Sun B., Notten P.H.L., Wang G. (2022). Oxygen redox chemistry in lithium-rich cathode materials for Li-ion batteries: Understanding from atomic structure to nano-engineering. Nano Mater. Sci..

[94] Li Q., Cao Z., Wahyudi W., Liu G., Park G.-T., Cavallo L., Anthopoulos T.D., Wang L., Sun Y.-K., Alshareef H.N. (2021). Unraveling the New Role of an Ethylene Carbonate Solvation Shell in Rechargeable Metal Ion Batteries. ACS Energy Lett..

[95] Yang J., Liu X., Wang Y., Zhou X., Weng L., Liu Y., Ren Y., Zhao C., Dahbi M., Alami J. (2021). Electrolytes Polymerization-Induced Cathode-Electrolyte-Interphase for High Voltage Lithium-Ion Batteries. Adv. Energy Mater..

[96] Hou X., Li T., Qiu Y., Jiang M., Lin H., Zheng Q., Li X. (2024). Interfacial Chemistry of Perfluorinated-Anion Additives Deciphering Ether-Based Electrolytes for Sodium-Ion Batteries. ACS Energy Lett..

[97] Luo X., Xing L., Vatamanu J., Chen J., Chen J., Liu M., Wang C., Xu K., Li W. (2022). Inhibiting manganese (II) from catalyzing electrolyte decomposition in lithium-ion batteries. J. Energy Chem..

[98] Yang H., Wang D., Liu Y., Liu Y., Zhong B., Song Y., Kong Q., Wu Z., Guo X. (2024). Improvement of cycle life for layered oxide cathodes in sodium-ion batteries. Energy Environ. Sci..

[99] Yu T.-Y., Ryu H.-H., Han G., Sun Y.-K. (2020). Understanding the Capacity Fading Mechanisms of O3-Type Na[Ni_0.5_Mn_0.5_]O_2_ Cathode for Sodium-Ion Batteries. Adv. Energy Mater..

[100] Li F., Liu Z., Liao C., Xu X., Zhu M., Liu J. (2023). Gradient boracic polyanion doping-derived surface lattice modulation of high-voltage Ni-rich layered cathodes for high-energy-density Li-ion batteries. ACS Energy Lett..

[101] Feng L., Guo J., Sun C., Xiao X., Feng L., Hao Y., Sun G., Tian Z., Li T., Li Y. (2024). An Active Strategy to Reduce Residual Alkali for High-Performance Layered Oxide Cathode Materials of Sodium-Ion Batteries. Small.

[102] Zhang T., Kong J., Shen C., Cui S., Lin Z., Deng Y., Song M., Jiao L., Huang H., Jin T. (2023). Converting Residual Alkali into Sodium Compensation Additive for High-Energy Na-Ion Batteries. ACS Energy Lett..

[103] Jo C.-H., Cho D.-H., Noh H.-J., Yashiro H., Sun Y.-K., Myung S.T. (2015). An effective method to reduce residual lithium compounds on Ni-rich Li[Ni_0.6_Co_0.2_Mn_0.2_]O_2_ active material using a phosphoric acid derived Li_3_PO_4_ nanolayer. Nano Res..

[104] Lux S.F., Lucas I.T., Pollak E., Passerini S., Winter M., Kostecki R. (2012). The mechanism of HF formation in LiPF_6_ based organic carbonate electrolytes. Electrochem. Commun..

[105] Åvall G., Adelhelm P. (2022). Solution to dissolution. Nat. Energy.

[106] Lin J., Peng H., Huang P., Naren T., Liang C., Kuang G., Chen L., Zhang C., Wei W. (2023). Electrically Coupled Electrolyte Engineering Enables High Interfacial Stability for High-Voltage Sodium-Ion Batteries. Adv. Funct. Mater..

[107] Ren Q., Yuan Y., Wang S. (2022). Interfacial Strategies for Suppression of Mn Dissolution in Rechargeable Battery Cathode Materials. ACS Appl. Mater. Interfaces.

[108] Cui T., Liu L., Xiang Y., Sheng C., Li X., Fu Y. (2024). Facilitating an Ultrastable O3-Type Cathode for 4.5 V Sodium-Ion Batteries via a Dual-Reductive Coupling Mechanism. J. Am. Chem. Soc..

[109] Dai P., Shi C.-G., Huang Z., Wu X.-H., Deng Y.-P., Fu J., Xie Y., Fan J., Shen S., Shen C.-H. (2023). A new film-forming electrolyte additive in enhancing the interface of layered cathode and cycling life of sodium ion batteries. Energy Storage Mater..

[110] Park J.-H., Park K., Kim R.-H., Yun D.-J., Park S.-Y., Han D., Lee S.-S., Park J.-H. (2015). Improving the kinetics and surface stability of sodium manganese oxide cathode materials for sodium rechargeable batteries with Al_2_O_3_/MWCNT hybrid networks. J. Mater. Chem. A.

[111] Su Y., Cui S., Zhuo Z., Yang W., Wang X., Pan F. (2015). Enhancing the High-Voltage Cycling Performance of LiNi_0.5_Mn_0.3_Co_0.2_O_2_ by Retarding Its Interfacial Reaction with an Electrolyte by Atomic-Layer-Deposited Al_2_O_3_. ACS Appl. Mater. Interfaces.

[112] Liu W., Li X., Xiong D., Hao Y., Li J., Kou H., Yan B., Li D., Lu S., Koo A. (2018). Significantly improving cycling performance of cathodes in lithium ion batteries: The effect of Al_2_O_3_ and LiAlO_2_ coatings on LiNi_0.6_Co_0.2_Mn_0.2_O_2_. Nano Energy.

[113] Zhang Y., Liu L., Jamil S., Xie J., Liu W., Xia J., Nie S., Wang X. (2019). Al_2_O_3_ coated Na_0.44_MnO_2_ as high-voltage cathode for sodium ion batteries. Appl. Surf. Sci..

[114] Liu Y., Fang X., Zhang A., Shen C., Liu Q., Enaya H.A., Zhou C. (2016). Layered P2-Na_2/3_[Ni_1/3_Mn_2/3_]O_2_ as high-voltage cathode for sodium-ion batteries: The capacity decay mechanism and Al_2_O_3_ surface modification. Nano Energy.

[115] Kalapsazova M., Kukeva R., Harizanova S., Markov P., Nihtianova D., Zhecheva E., Stoyanova R. (2023). High-Performance Layered Oxides for Sodium-Ion Batteries Achieved through Combined Aluminum Substitution and Surface Treatment. Batteries.

[116] Ji H., Zhai J., Chen G., Qiu X., Fang H., Zhang T., Huang Z., Zhao W., Wang Z., Chu M. (2022). Surface Engineering Suppresses the Failure of Biphasic Sodium Layered Cathode for High Performance Sodium-Ion Batteries. Adv. Funct. Mater..

[117] Zhang X., Belharouak I., Li L., Lei Y., Elam J.W., Nie A., Chen X., Yassar R.S., Axelbaum R.L. (2013). Structural and Electrochemical Study of Al_2_O_3_ and TiO_2_ Coated Li_1.2_Ni_0.13_Mn_0.54_Co_0.13_O_2_ Cathode Material Using ALD. Adv. Energy Mater..

[118] Kaliyappan K., Liu J., Xiao B., Lushington A., Li R., Sham T.-K., Sun X. (2017). Enhanced Performance of P2-Na_0.66_(Mn_0.54_Co_0.13_Ni_0.13_)O_2_ Cathode for Sodium-Ion Batteries by Ultrathin Metal Oxide Coatings via Atomic Layer Deposition. Adv. Funct. Mater..

[119] Zhao J., Wang Y. (2013). Atomic layer deposition of epitaxial ZrO_2_ coating on LiMn_2_O_4_ nanoparticles for high-rate lithium ion batteries at elevated temperature. Nano Energy.

[120] Song B., Li W., Oh S.-M., Manthiram A. (2017). Long-Life Nickel-Rich Layered Oxide Cathodes with a Uniform Li_2_ZrO_3_ Surface Coating for Lithium-Ion Batteries. ACS Appl. Mater. Interfaces.

[121] Yu Y., Ning D., Li Q., Franz A., Zheng L., Zhang N., Ren G., Schumacher G., Liu X. (2021). Revealing the anionic redox chemistry in O3-type layered oxide cathode for sodium-ion batteries. Energy Storage Mater..

[122] Yang Y., Dang R., Wu K., Li Q., Li N., Xiao X., Hu Z. (2020). Semiconductor Material ZnO-Coated P2-Type Na2/3Ni1/3Mn2/3O2 Cathode Materials for Sodium-Ion Batteries with Superior Electrochemical Performance. J. Phys. Chem. C.

[123] Hwang J.-Y., Yu T.-Y., Sun Y.-K. (2018). Simultaneous MgO coating and Mg doping of Na[Ni_0.5_Mn_0.5_]O_2_ cathode: Facile and customizable approach to high-voltage sodium-ion batteries. J. Mater. Chem. A.

[124] Liu H., Wang Y., Wang Y., Safdar A., Wu F., Gao H. (2024). Exploring the Effect of TiO_2_ Coating on Na_0.85_Li_0.12_Ni_0.22_Mn_0.66_O_2_ Cathode Materials for Na-Ion Batteries. J. Electrochem. Soc..

[125] Zhou L., Zhang Z., Lv S., Zhang M., Jiao P., Zhang W., Xu J., Zhang K. (2023). SnO_2_ coating to stabilize Mn-based layered oxide cathode materials for sodium-ion batteries. Mater. Today Energy.

[126] Li B., Wang J., Cao Z., Zhang P., Zhao J. (2016). The role of SnO_2_ surface coating in the electrochemical performance of Li_1.2_Mn_0.54_Co_0.13_Ni_0.13_O_2_ cathode materials. J. Power Sources.

[127] Chen C., Geng T., Du C., Zuo P., Cheng X., Ma Y., Yin G. (2016). Oxygen vacancies in SnO2 surface coating to enhance the activation of layered Li-Rich Li_1.2_Mn_0.54_Ni_0.13_Co_0.13_O_2_ cathode material for Li-ion batteries. J. Power Sources.

[128] Kalapsazova M., Kukeva R., Zhecheva E., Stoyanova R. (2022). Metal Substitution versus Oxygen-Storage Modifier to Regulate the Oxygen Redox Reactions in Sodium-Deficient Three-Layered Oxides. Batteries.

[129] Jo J.H., Choi J.U., Konarov A., Yashiro H., Yuan S., Shi L., Sun Y.-K., Myung S.-T. (2018). Sodium-Ion Batteries: Building Effective Layered Cathode Materials with Long-Term Cycling by Modifying the Surface via Sodium Phosphate. Adv. Funct. Mater..

[130] Xu W., Dang R., Zhou L., Yang Y., Lin T., Guo Q., Xie F., Hu Z., Ding F., Liu Y. (2023). Conversion of Surface Residual Alkali to Solid Electrolyte to Enable Na-Ion Full Cells with Robust Interfaces. Adv. Mater..

[131] Jo C.-H., Jo J.-H., Yashiro H., Kim S.-J., Sun Y.-K., Myung S.-T. (2018). Bioinspired Surface Layer for the Cathode Material of High-Energy-Density Sodium-Ion Batteries. Adv. Energy Mater..

[132] Wang H., Ding F., Wang Y., Han Z., Dang R., Yu H., Yang Y., Chen Z., Li Y., Xie F. (2023). In Situ Plastic-Crystal-Coated Cathode toward High-Performance Na-Ion Batteries. ACS Energy Lett..

[133] Tang K., Huang Y., Xie X., Cao S., Liu L., Liu M., Huang Y., Chang B., Luo Z., Wang X. (2020). The effects of dual modification on structure and performance of P2-type layered oxide cathode for sodium-ion batteries. Chem. Eng. J..

[134] Deng Q., Zheng F., Zhong W., Pan Q., Liu Y., Li Y., Li Y., Hu J., Yang C., Liu M. (2021). Nanoscale surface modification of P2-type Na_0.65_[Mn_0.70_Ni_0.16_Co_0.14_]O_2_ cathode material for high-performance sodium-ion batteries. Chem. Eng. J..

[135] Sun H.-H., Hwang J.-Y., Yoon C.S., Heller A., Mullins C.B. (2018). Capacity Degradation Mechanism and Cycling Stability Enhancement of AlF_3_-Coated Nanorod Gradient Na[Ni_0.65_Co_0.08_Mn_0.27_]O_2_ Cathode for Sodium-Ion Batteries. ACS Nano.

[136] Feng S., Lu Y., Lu X., Chen H., Wu X., Wu M., Xu F., Wen Z. (2024). Surface Engineering through In Situ Construction of Co_x_B-Spinel Dual Coating Layers for High-Voltage Stable Sodium-Ion Batteries. Adv. Energy Mater..

[137] Huang Y., Zhang W., Zhou Y., Wang Y., Li L., Shao H., Li X., Hong Z., Xia H., Shen Y. (2024). Air Corrosion of Layered Cathode Materials for Sodium-Ion Batteries: Cation Mixing and a Practical Suppression Strategy. ACS Nano.

[138] Wang C., Wang X., Zou P., Zhang R., Wang S., Song B., Low K.-B., Xin H.L. (2023). Direct observation of chemomechanical stress-induced phase transformation in high-Ni layered cathodes for lithium-ion batteries. Matter.

[139] Kim D.-H., Song J.-H., Jung C.-H., Eum D., Kim B., Hong S.-H., Kang K. (2022). Stepwise Dopant Selection Process for High-Nickel Layered Oxide Cathodes. Adv. Energy Mater..

[140] Wang K., Wan H., Yan P., Chen X., Fu J., Liu Z., Deng H., Gao F., Sui M. (2019). Dopant Segregation Boosting High-Voltage Cyclability of Layered Cathode for Sodium Ion Batteries. Adv. Mater..

[141] Wang L., Sun M.-Y., Deng L., Zheng Y.-Q., Li X.-Y., Jiang Y.-S., Zhao L., Wang Z.-B. (2024). Ti^4+^ substitution suppressing P2-O2 phase transition to construct stable P2-Na_0.67_Ni_0.33_Mn_0.67_O_2_ cathode for long-term durable sodium-ion batteries. J. Energy Storage.

[142] Tang Y., Zhang Q., Zuo W., Zhou S., Zeng G., Zhang B., Zhang H., Huang Z., Zheng L., Xu J. (2024). Sustainable layered cathode with suppressed phase transition for long-life sodium-ion batteries. Nat. Sustain..

[143] Shen Q., Liu Y., Zhao X., Jin J., Wang Y., Li S., Li P., Qu X., Jiao L. (2021). Transition-Metal Vacancy Manufacturing and Sodium-Site Doping Enable a High-Performance Layered Oxide Cathode through Cationic and Anionic Redox Chemistry. Adv. Funct. Mater..

[144] Yang L., Li X., Liu J., Xiong S., Ma X., Liu P., Bai J., Xu W., Tang Y., Hu Y.-Y. (2019). Lithium-Doping Stabilized High-Performance P2–Na_0.66_Li_0.18_Fe_0.12_Mn_0.7_O_2_ Cathode for Sodium Ion Batteries. J. Am. Chem. Soc..

[145] Wang X., Zhang Q., Zhao C., Li H., Zhang B., Zeng G., Tang Y., Huang Z., Hwang I., Zhang H. (2024). Achieving a high-performance sodium-ion pouch cell by regulating intergrowth structures in a layered oxide cathode with anionic redox. Nat. Energy.

[146] Li N., Zhao E., Zhang Z., Yin W., He L., Wang B., Wang F., Xiao X., Zhao J. (2024). Gradient and De-Clustered Anionic Redox Enabled Undetectable O2 Formation in 4.5 V Sodium Manganese Oxide Cathodes. Adv. Mater..

[147] Sun L., Wu Z., Hou M., Ni Y., Sun H., Jiao P., Li H., Zhang W., Zhang L., Zhang K. (2024). Unraveling and suppressing the voltage decay of high-capacity cathode materials for sodium-ion batteries. Energy Environ. Sci..

[148] Zhang T., Ren M., Huang Y., Li F., Hua W., Indris S., Li F. (2024). Negative Lattice Expansion in an O3-Type Transition-Metal Oxide Cathode for Highly Stable Sodium-Ion Batteries. Angew. Chem. Int. Ed..

[149] Li Y., Mazzio K.A., Yaqoob N., Sun Y., Freytag A.I., Wong D., Schulz C., Baran V., Mendez A.S.J., Schuck G. (2024). Competing Mechanisms Determine Oxygen Redox in Doped Ni-Mn Based Layered Oxides for Na-Ion Batteries. Adv. Mater..

[150] Kim S., Park S.O., Lee M.-Y., Lee J.-A., Kristanto I., Lee T.K., Hwang D., Kim J., Wi T.-U., Lee H.-W. (2022). Stable electrode–electrolyte interfaces constructed by fluorine- and nitrogen-donating ionic additives for high-performance lithium metal batteries. Energy Storage Mater..

[151] Zhou X., Chen X., Yang Z., Liu X., Hao Z., Jin S., Zhang L., Wang R., Zhang C., Li L. (2024). Anion Receptor Weakens ClO_4_^−^ Solvation for High-Temperature Sodium-Ion Batteries. Adv. Funct. Mater..

[152] Zheng J., Chen S., Zhao W., Song J., Engelhard M.H., Zhang J.-G. (2018). Extremely Stable Sodium Metal Batteries Enabled by Localized High-Concentration Electrolytes. ACS Energy Lett..

[153] Liu Q., Feng Y.-H., Zhu X., Liu M., Yu L., Wei G.-X., Fan X.-Y., Ji X., Wang P.-F., Xin H. (2024). Stabilizing cathode-electrolyte interphase by localized high-concentration electrolytes for high-voltage sodium-ion batteries. Nano Energy.

[154] Fan J.-J., Dai P., Shi C.-G., Wen Y., Luo C.-X., Yang J., Song C., Huang L., Sun S.-G. (2021). Synergistic Dual-Additive Electrolyte for Interphase Modification to Boost Cyclability of Layered Cathode for Sodium Ion Batteries. Adv. Funct. Mater..

[155] He J., Bhargav A., Su L., Lamb J., Okasinski J., Shin W., Manthiram A. (2024). Tuning the solvation structure with salts for stable sodium-metal batteries. Nat. Energy.

[156] Jin Y., Le P.M.L., Gao P., Xu Y., Xiao B., Engelhard M.H., Cao X., Vo T.D., Hu J., Zhong L. (2022). Low-solvation electrolytes for high-voltage sodium-ion batteries. Nat. Energy.

[157] Sun M.-Y., Liu B., Xia Y., Wang Y.-X., Zheng Y.-Q., Wang L., Deng L., Zhao L., Wang Z.-B. (2024). Reorganizing Helmholtz Adsorption Plane Enables Sodium Layered-Oxide Cathode beyond High Oxidation Limits. Adv. Mater..

[158] Zhang H., Xu X., Fan W., Zhao J., Huo Y. (2024). In-Situ Polymerized Solid/Quasi-Solid Polymer Electrolyte for Lithium-Metal Batteries: Recent Progress and Perspectives. Chem. Eur. J..

[159] Yang Y., Yang S., Xue X., Zhang X., Li Q., Yao Y., Rui X., Pan H., Yu Y. (2024). Inorganic All-Solid-State Sodium Batteries: Electrolyte Designing and Interface Engineering. Adv. Mater..

[160] Wu E.A., Banerjee S., Tang H., Richardson P.M., Doux J.-M., Qi J., Zhu Z., Grenier A., Li Y., Zhao E. (2021). A stable cathode-solid electrolyte composite for high-voltage, long-cycle-life solid-state sodium-ion batteries. Nat. Commun..

[161] Lin X., Zhang S., Yang M., Xiao B., Zhao Y., Luo J., Fu J., Wang C., Li X., Li W. (2024). A family of dual-anion-based sodium superionic conductors for all-solid-state sodium-ion batteries. Nat. Mater..

